# Supplementary material for: Analysis of cardiac magnetic resonance imaging in 36,000 individuals yields genetic insights into dilated cardiomyopathy
Source: Nat Commun. 2020 May 7;11:2254. doi: 10.1038/s41467-020-15823-7 (PMC7206184; doi:10.1038/s41467-020-15823-7)
Supplement: Supplementary file 1 — Supplementary Information [file 41467_2020_15823_MOESM1_ESM.pdf]

Supplementary Information

Pirruccello, *et al*, "Analysis of cardiac magnetic resonance imaging in 36,000 individuals yields genetic insights into dilated cardiomyopathy"

Supplementary Index

Supplementary Index 1

Supplementary Notes 3

Supplementary Notes 1: Sensitivity analysis in Europeans 3

Supplementary Methods 4

Sensitivity analysis in Europeans 4

MESA cardiac magnetic resonance imaging 5

MESA whole genome sequencing 6

Supplementary Tables 6

Supplementary Table 1: Loci significant at various P value thresholds 6

Supplementary Table 2: LD score regression 6

Supplementary Table 3: Lookup of SNPs from prior GWAS studies in the cardiac MRI GWAS 7

Supplementary Table 4: Characteristics of MESA Participants 8

Supplementary Table 5: Genes in cardiomyopathy panels 9

Supplementary Table 6: Colocation with Mendelian cardiomyopathy genes 10

Supplementary Table 7: PheWAS results for curated disease phenotypes 10

Supplementary Table 8: Characteristics of unrelated participants who did not undergo cardiac MRI 11

Supplementary Table 9: Polygenic scores from cardiac MRI phenotypes are associated with incident DCM 13

Supplementary Table 10: Characteristics of TTNtv carriers 13

Supplementary Table 11: Imputed TTNtv were not confirmed by exome sequencing 14

Supplementary Table 12: Clinical characteristics of European UK Biobank participants with cardiac MRI data 14

Supplementary Table 13: Heritability in the full sample and in the European-specific subset 16

Supplementary Table 14: LD score regression in the European-specific analysis 16

Supplementary Table 15: Loci significant at various P value thresholds in the European-specific analysis 17

Supplementary Table 16: Loci newly achieving genome-wide significance in the European-specific analysis 17
Supplementary Table 17: Concordance of effect estimates from lead SNPs in the main analysis and the European-specific analysis 18
Supplementary Table 18: Directional concordance of genome-wide significant SNPs from the main analysis and the European-specific analysis 20
Supplementary Table 19: Characteristics of unrelated European participants who did not undergo cardiac MRI 20
Supplementary Table 20: Polygenic scores from cardiac MRI phenotypes are associated with incident DCM in the European-specific analysis 21
Supplementary Figures 22
Supplementary Figure 1: Flow diagram for UK Biobank GWAS participants 22
Supplementary Figure 2: Sex-stratified distributions of cardiac MRI phenotypes 23
Supplementary Figure 3: Cross-trait correlation 24
Supplementary Figure 4: Genetic correlation 25
Supplementary Figure 5: MAGMA gene expression enrichment 26
LVEDV 26
LVESV 27
LVEF 27
SV 28
LVEDVi 28
LVESVi 29
SVi 29
Supplementary Figure 6: GWAS loci are found nearby to more cardiomyopathy-related genes than expected by chance 30
Supplementary Figure 7: PheWAS 32
Supplementary Figure 8: SNP effect estimates in the main analysis and in the European-specific analysis 35
Supplementary References 36

Supplementary Notes

Supplementary Notes 1: Sensitivity analysis in Europeans

Because we ran BOLT with the default European linkage disequilibrium panel for the main analyses, we also conducted sensitivity analyses using a subset of rigorously-defined European samples to understand whether our results were influenced by residual population stratification. To do this, we defined a genetically similar subset of participants who self-described as British, Irish, or another European ancestry and underwent cardiac MRI (N = 32,755, 9% fewer samples than in the main analysis). The characteristics of these participants are described in **Supplementary Table 12**.

The heritability estimates for each cardiac trait in the European-specific analysis were similar to those in the main analysis (**Supplementary Table 13**). The GWAS in Europeans showed similar evidence for polygenicity, and lack of confounding, as were observed in the main analysis (**Supplementary Table 14**).

Eight fewer loci achieved genome-wide significance in the European-specific analysis (49 distinct loci instead of 57 in the main analysis, or fewer loci with more stringent P value thresholds as in **Supplementary Table 15**). Four loci newly achieved genome-wide significance in the European-specific analysis (**Supplementary Table 16**). These include loci near *ARPC1A*, whose gene product is actin related protein 2/3 complex subunit 1A; *PDGFD*, whose gene product is platelet derived growth factor D; *LSM7*; and *LYN*. Twelve of the lead SNPs identified

in the main analysis with $P < 5\times10^{-8}$ did not have $P < 5\times10^{-8}$ in the European-specific analysis for any trait, although all SNPs with $P < 5\times10^{-8}$ in the main analysis had association $P < 3\times10^{-6}$ or stronger in the European-specific analysis. All lead SNPs from the main analysis are shown with their corresponding European-specific effect size and P value in **Supplementary Data File 6**.

Across various P value thresholds, the effect estimates for each SNP showed strong correlation with those in the main analysis, with nearly perfect correlation for all SNPs beyond $P < 5\times10^{-6}$ (**Supplementary Table 17**, **Supplementary Figure 8**). Of the SNPs with $P < 5\times10^{-8}$ in the main analysis, none had an effect in the opposite direction in the European-specific analysis (**Supplementary Table 18**).

Polygenic scores produced using the same procedure as in the main analysis yielded similar hazard ratios (participant characteristics in **Supplementary Table 19** and results in **Supplementary Table 20**). The best performing score, derived from the LVESVi lead SNPs, yielded an HR of 1.56 for incident dilated cardiomyopathy in the European-only subset, similar to the HR of 1.58 from the polygenic score produced in the main analysis.

Supplementary Methods

Sensitivity analysis in Europeans

Starting with participants in the UK Biobank who self-identified as British, Irish, or Other European, we applied the *aberrant* R package as previously described[1,2]. Briefly, we used the first 6 principal components (PCs) of ancestry in pairs (PC 1 and 2; PC 3 and 4; PC 5 and 6)

with lambda (the ratio of standard deviations of outliers vs inliers) set to 40. We took the intersection of these 3 pairs, isolating a tight genetic inlier cluster of European participants.

For each trait, we then conducted a heritability analysis with BOLT-REML and a GWAS with BOLT-LMM. Using the lead SNPs from this smaller European sample, we produced polygenic scores and applied them in the European subset of the remaining unrelated UK Biobank participants who had not undergone cardiac MRI. Aside from using this genetically defined subset of European participants, all analyses were conducted in the same fashion and with the same settings as in the main analysis.

To evaluate the SNP-level differences in the GWAS, we compared effect estimates (Beta) for each SNP in the main analysis with those in the European-specific analysis using a linear model. Taking SNPs below a given P value threshold in the main analysis, agreement was assessed by the overall model fit (r^2). This procedure was performed for each trait across several P value thresholds.

MESA cardiac magnetic resonance imaging

MESA is a longitudinal cohort study of cardiovascular disease in 6,814 participants aged 44-84 years at enrollment and recruited in six US sites. A baseline examination was held from 2000-2002 and follow up exams from 2002 – 2012[3]. Cardiovascular MRI was performed on 5,0004 participants at baseline and 3,015 during a follow-up examination (“Exam 5”). The MRI protocol during Exam 5 used a similar technique (steady-state free precession) to that used in the UK Biobank, and was therefore chosen for comparison against the same 7 traits in the main analysis.

MESA whole genome sequencing

Whole genome sequencing and quality control of the MESA participants was described previously[4]. In brief, ~4,610 MESA participants were chosen for whole genome sequencing at the Broad Institute (Cambridge, MA), with written consent and IRB approval (IRB #2016P001308). Sequencing was performed on Illumina HiSeqX platform with 151-bp paired-end reads. Samples were excluded from downstream variant calling if the estimated contamination was >= 3% or <95% of the genome was covered to at least 10x read depth. Variants were called with GATK version 3 HaplotypeCaller[5].

Supplementary Tables

Supplementary Table 1: Loci significant at various P value thresholds

P threshold	LVEDV	LVEDVi	LVESV	LVESVi	LVEF	SV	SVi	Total distinct loci
5.00E-08	22	14	22	32	28	12	8	57
1.00E-08	17	7	15	23	21	9	5	37
5.00E-09	17	7	15	21	19	9	3	36
1.00E-09	14	5	14	17	15	5	2	28

“P threshold” represents the P value cutoff. “Total distinct loci” is a count of unique loci aggregated across all traits, so that any locus found in two or more traits is only counted once.

Supplementary Table 2: LD score regression

	Lambda	Intercept
**LVEDV**	1.1459	1.0187
**LVEDVi**	1.1459	0.9976
**LVESV**	1.1459	1.0078

**LVESVi**	1.1459	0.9917
**LVEF**	1.0957	0.9952
**SV**	1.1459	1.0204
**SVi**	1.0957	1.0067

Lambda represents the genomic inflation factor. Intercept represents the intercept from LD score regression.

Supplementary Table 3: Lookup of SNPs from prior GWAS studies in the cardiac MRI GWAS

Study	Discovery Trait	SNP	CHR	BP_HG19	Nearby Genes	Locus	Discovery P	MRI Trait	MRI GWAS P
Kanai	LVIDd	rs34866937	8	125859850	MTSS1	TTE 1	1.77E-09	LVEDV	6.20E-11
Kanai	LVIDd	rs3812625	10	75757702	VCL	TTE 2	1.42E-08	LVEDV	6.00E-01
Kanai	LVIDd	rs11874741	18	30077859	GAREM1	TTE 3	4.94E-08	LVEDV	9.70E-01
Kanai	LVIDs	rs6546120	2	65238407	SLC1A4	TTE 4	4.79E-08	LVESV	4.90E-01
Kanai	LVIDs	rs34866937	8	125859850	MTSS1	TTE 1	1.60E-13	LVESV	8.70E-13
Kanai	LVIDs	rs11874741	18	30077859	GAREM1	TTE 3	4.62E-08	LVESV	9.90E-01
Kanai	LVIDs	rs5760061	22	24178279	SMARCB1/DERL3	TTE 5	8.63E-11	LVESV	1.30E-13
Kanai	Fractional Shortening	rs6546120	2	65238407	SLC1A4	TTE 4	1.70E-08	LVEF	7.20E-01
Kanai	Fractional Shortening	rs34866937	8	125859850	MTSS1	TTE 1	2.40E-13	LVEF	1.40E-07
Kanai	Fractional Shortening	rs11025521	11	20370206	DBX1	TTE 6	4.41E-08	LVEF	6.80E-02
Kanai	Fractional Shortening	rs5760054	22	24161717	SMARCB1/DERL3	TTE 5	1.01E-11	LVEF	2.70E-13
Kanai	Ejection Fraction	rs6546120	2	65238407	SLC1A4	TTE 4	3.35E-08	LVEF	7.20E-01
Kanai	Ejection Fraction	rs34866937	8	125859850	MTSS1	TTE 1	8.97E-16	LVEF	1.40E-07
Kanai	Ejection Fraction	rs5760061	22	24178279	SMARCB1/DERL3	TTE 5	6.84E-11	LVEF	2.80E-13
Wild	LVIDd	rs11153730	6	118667522	SLC35F1/PLN	TTE 7	6.40E-16	LVEDV	3.70E-16
Wild	LVIDd	rs12541595	8	125857359	MTSS1	TTE 1	3.02E-12	LVEDV	1.80E-10
Wild	LVIDd	rs10774625	12	111910219	ATXN2	TTE 8	1.90E-08	LVEDV	1.70E-15
Wild	Fractional Shortening	rs9470361	6	36623379	CDKN1A	TTE 9	5.30E-09	LVEF	2.00E-15
Wild	LV Mass	rs1454157	4	177358798	SPCS3	TTE 10	4.41E-09		
Esslinger	DCM	rs10927875	1	16299312	CLCNKA	DCM 1	8.11E-13	LVEF	3.10E-21
Esslinger	DCM	rs848210	1	16259813	CLCNKA	DCM 1	6.30E-07	LVEF	4.30E-20
Esslinger	DCM	rs3829746	2	179427536	TTN	DCM 2	3.40E-07	LVESV	2.30E-19
Esslinger	DCM	rs13107325	4	103188709	SLC39A8	DCM 3	6.00E-07	LVESV	1.10E-04
Esslinger	DCM	rs4712056	6	53989526	MLIP	DCM 4	5.10E-07	LVEDV	1.50E-06
Esslinger	DCM	rs2291569	7	128488734	FLNC	DCM 5	8.70E-11	LVESV	2.80E-10
Esslinger	DCM	rs2234962	10	121429633	BAG3	DCM 6	1.70E-25	LVESV	1.80E-30
Esslinger	DCM	rs3188055	10	121586882	BAG3	DCM 6	1.10E-08	LVEF	8.60E-06
Esslinger	DCM	rs1051168	15	85200520	ALPK3	DCM 7	4.10E-07	LVESVi	6.90E-10
Esslinger	DCM	rs3803403	15	85383145	ALPK3	DCM 7	2.90E-07	LVESV	1.20E-11
Esslinger	DCM	rs2303510	18	34324091	FHOD3	DCM 8	1.50E-07	LVEF	6.10E-06 [a]
Meder	DCM	rs9262636	6	31025848	HCG22	DCM 9	4.90E-09	LVEDVi	4.30E-01
Villard	DCM	rs10927875	1	16299312	CLCNKA	DCM 1	1.30E-07	LVEF	3.10E-21
Villard	DCM	rs2234962	10	121429633	BAG3	DCM 6	8.80E-10	LVEF	5.40E-31
Aung	LVEDV	rs2042995	2	179558366	TTN	AUN 1	2.30E-11	LVEDV	1.50E-14
Aung	LVEDV	rs7071853	10	121311606	BAG3	AUN 2	3.90E-09	LVEDV	1.70E-05
Aung	LVEDV	rs7310615	12	111865049	SH2B3	AUN 3	1.40E-09	LVEDV	4.90E-16
Aung	LVESV	rs2042995	2	179558366	TTN	AUN 4	8.40E-20	LVESV	5.90E-23
Aung	LVESV	rs200712209	8	125858538	MTSS1	AUN 5	1.70E-11	LVESV	2.70E-13
Aung	LVESV	rs72840788	10	121415685	BAG3	AUN 6	5.60E-17	LVESV	8.10E-32
Aung	LVEF	rs945425	1	16348412	CLCNKA	AUN 7	8.60E-11	LVEF	6.00E-25
Aung	LVEF	rs2042995	2	179558366	TTN	AUN 8	2.50E-12	LVEF	1.30E-18
Aung	LVEF	rs34866937	8	125859850	MTSS1	AUN 9	6.80E-11	LVEF	1.40E-07

Aung	LVEF	rs72840788	10	121415685	BAG3	AUN 10	3.40E-15	LVEF	4.00E-32
Aung	LV Mass	rs2255167	2	179558282	TTN	AUN 11	8.30E-14		
Aung	LV Mass-Volume Ratio	rs146170154	6	36646768	CDKN1A	AUN 12	2.60E-11		
Aung	LV Mass-Volume Ratio	rs149369954	15	85348961	ZNF592/ALPK3	AUN 13	1.90E-11		
Aung	LV Mass-Volume Ratio	rs6003909	22	24181652	DERL3	AUN 14	9.70E-15		

Study: The discovery study in which the SNPs were identified: Kanai, Wild, Esslinger, Meder, VIllard, Aung[6–11]. LVIDd: left ventricular internal diameter at end diastole from transthoracic echocardiography (TTE). LVIDs: left ventricular diameter at end systole from TTE. LV Mass: left ventricular mass from TTE. DCM: dilated cardiomyopathy. The “Locus” column tracks unique genomic loci, such that, within a study type (TTE or DCM), SNPs within 1 megabase of one another are given the same Locus identifier. For SNPs from the studies of cardiac traits, the most similar MRI trait was used for comparison. No similar trait was available for comparison to LV Mass. For SNPs from the DCM GWAS studies, the MRI trait with the lowest P-value was taken for each SNP. The “MRI GWAS P” column represents the association P value of the SNP from the SNP column within in the cardiac MRI GWAS from the “MRI Trait” column. The MRI GWAS P value column is shaded blue if the SNP had $P < 5 \times 10^{-8}$ in the MRI GWAS, and yellow if the SNP had $5 \times 10^{-8} < P < 1 \times 10^{-3}$. The values represent the P value for the specific SNP, but other SNPs in linkage disequilibrium may have stronger association P values. Note: (a) At rs2303510 (DCM 8), the nearby SNP rs2047273 ($r^2 = 0.29$) in the locus had an association P = 3.1×10^{-8} with LVEDV. After accounting for SNPs in linkage disequilibrium, five of the 10 TTE loci and six of the nine DCM loci have a genome-wide significant SNP in the cardiac MRI GWAS.

Supplementary Table 4: Characteristics of MESA Participants

		**Women**	**Men**	**All**
		1204	980	2184
**Age at MRI**				
	Mean (SD)	69 (9.1)	68 (8.9)	69 (9.1)
	Median [Q1-Q3]	68 [61 - 76]	67 [61 - 75]	68 [61 - 75]
	Missing	5 (0.4%)	3 (0.3%)	8 (0.4%)
**Site**				
	Wake Forest	197 (16 %)	135 (14 %)	332 (15 %)
	Columbia	200 (17 %)	165 (17 %)	365 (17 %)
	Johns Hopkins	181 (15 %)	127 (13 %)	308 (14 %)
	Minnesota	173 (14 %)	160 (16 %)	333 (15 %)
	Northwestern	243 (20 %)	186 (19 %)	429 (20 %)
	UCLA	210 (17 %)	207 (21 %)	417 (19 %)
**Ancestry**				
	European	537 (45 %)	403 (41 %)	940 (43 %)
	East Asian	156 (13 %)	143 (15 %)	299 (14 %)
	African-American	297 (25 %)	210 (21 %)	507 (23 %)
	Hispanic	214 (18 %)	224 (23 %)	438 (20 %)
**BMI (kg/cm^2)**				
	Mean (SD)	28 (5.4)	27 (4.0)	28 (4.8)
	Median [Q1-Q3]	27 [24 - 31]	27 [25 - 30]	27 [24 - 30]
**Weight (kg)**				

	Mean (SD)	72 (15)	83 (14)	77 (16)
	Median [Q1-Q3]	69 [61 - 81]	83 [73 - 92]	75 [65 - 87]
**Height (cm)**				
	Mean (SD)	160 (6.8)	170 (7.6)	170 (9.7)
	Median [Q1-Q3]	160 [160 - 170]	170 [170 - 180]	170 [160 - 170]
**LVEDV**				
	Mean (SD)	110 (23)	130 (32)	120 (31)
	Median [Q1-Q3]	100 [90 - 120]	130 [110 - 150]	110 [96 - 140]
**LVEDVi**				
	Mean (SD)	59 (11)	67 (14)	63 (13)
	Median [Q1-Q3]	59 [52 - 66]	66 [57 - 76]	62 [54 - 70]
**LVESV**				
	Mean (SD)	38 (12)	54 (18)	45 (17)
	Median [Q1-Q3]	36 [30 - 45]	51 [41 - 63]	42 [33 - 54]
**LVESVi**				
	Mean (SD)	21 (6.0)	27 (8.1)	24 (7.5)
	Median [Q1-Q3]	21 [17 - 25]	26 [21 - 31]	23 [19 - 28]
**LVEF**				
	Mean (SD)	64 (6.6)	60 (6.7)	62 (7.0)
	Median [Q1-Q3]	65 [60 - 69]	60 [56 - 65]	63 [58 - 67]
**SV**				
	Mean (SD)	67 (15)	80 (19)	73 (18)
	Median [Q1-Q3]	67 [57 - 76]	78 [66 - 93]	71 [61 - 84]
**SVi**				
	Mean (SD)	38 (7.5)	40 (9.1)	39 (8.3)
	Median [Q1-Q3]	38 [33 - 43]	40 [34 - 46]	39 [33 - 44]

MESA participants with both whole genome sequencing data and cardiac MRI at “Exam 5” were included in the analysis, excluding those with heart disease or evidence of myocardial scar.

Supplementary Table 5: Genes in cardiomyopathy panels

The 129 genes in this table were assembled from 3 commercially available cardiomyopathy gene panels noted in the main text: from GeneDx, Invitae, and the Partners Laboratory for Molecular Medicine.

A2ML1	CALR3	DOLK	GAA	LAMP2	MT-TH	MYH7	PDLIM3	SCN5A	TMEM70
ABCC9	CASQ2	DSC2	GATA4	LDB3	MT-TI	MYL2	PKP2	SDHA	TMPO
ACADVL	CAV3	DSG2	GATA6	LMNA	MT-TK	MYL3	PLEKHM2	SGCD	TNNC1
ACTC1	CBL	DSP	GATAD 1	LRRC10	MT-TL1	MYLK2	PLN	SHOC2	TNNI3
ACTN2	CHRM2	DTNA	GLA	MAP2K1	MT-TL2	MYOM1	PRDM16	SLC22A5	TNNT2
AGL	CPT2	ELAC2	HCN4	MAP2K2	MT-TM	MYOZ2	PRKAG2	SOS1	TOR1AIP1
AKAP9	CRYAB	EMD	HFE	MED12	MT-TQ	MYPN	PTPN11	SOS2	TPM1
ALMS1	CSRP3	EYA4	HRAS	MIB1	MT-TS1	NEBL	RAF1	SPRED1	TRDN

ALPK3	CTF1	FHL1	ILK	MT-ND1	MT-TS2	NEXN	RASA1	TAZ	TTN
ANKRD1	CTNNA3	FHL2	JPH2	MT-ND5	MTO1	NF1	RBM20	TBX20	TTR
BAG3	DES	FKRP	JUP	MT-ND6	MURC	NKX2-5	RIT1	TCAP	TXNRD2
BRAF	DMD	FKTN	KRAS	MT-TD	MYBPC3	NPPA	RRAS	TGFB3	VCL
CACNA1C	DNAJC19	FLNC	LAMA4	MT-TG	MYH6	NRAS	RYR2	TMEM43	

Supplementary Table 6: Colocation with Mendelian cardiomyopathy genes

ACTN2	FLNC	NKX2-5	RBM20	TTN
ALPK3	GATA4	PLEKHM2	RYR2	
BAG3	MYH6	PLN	SHOC2	
CSRP3	MYH7	PTPN11	TMEM43	

Each listed gene is located within 500 kilobases of a lead SNP from one of the seven traits, and was found in the list of 129 Mendelian cardiomyopathy-related genes in **Supplementary Table 5**.

Supplementary Table 7: PheWAS results for curated disease phenotypes

Curated Disease Phenotype	PRS	N	N With Phenotype	Beta	SE	-log10(P)
Dilated cardiomyopathy	lvesvi	449027	923	0.413	0.034	33.07
Hypertension	lvedv	449027	156957	-0.039	0.003	31.01
Dilated cardiomyopathy	lvesv	449027	923	0.386	0.034	29.93
Dilated cardiomyopathy	lvef	449027	923	-0.384	0.034	28.01
Hypertension	sv	449027	156957	-0.036	0.003	27.20
Heart_Failure	lvesvi	449027	9226	0.109	0.011	23.52
Hypothyroidism	lvedvi	449027	28703	-0.059	0.006	20.63
Hypothyroidism	sv	449027	28703	-0.059	0.006	20.39
Hypertrophic cardiomyopathy	lvesvi	449027	357	-0.450	0.051	18.15
Hypothyroidism	lvesv	449027	28703	-0.054	0.006	17.52
Hypertrophic cardiomyopathy	lvef	449027	357	0.451	0.052	17.52
Psoriasis	lvedv	449027	7008	-0.100	0.012	16.31
Heart_Failure	lvef	449027	9226	-0.090	0.011	15.96
Psoriasis	sv	449027	7008	-0.097	0.012	15.30
Coronary_Artery_Disease	sv	449027	25195	-0.052	0.007	14.24
Hypertrophic cardiomyopathy	lvesv	449027	357	-0.395	0.053	13.26
Heart_Failure	lvesv	449027	9226	0.077	0.011	12.33
Hypercholesterolemia	sv	449027	85925	-0.029	0.004	12.15
Venous_thromboembolism	svi	449027	16854	0.057	0.008	11.68
Myocardial_Infarction	sv	449027	19286	-0.049	0.008	10.24

Hypertension	lvedvi	449027	156957	-0.021	0.003	9.95
Hypercholesterolemia	lvedv	449027	85925	-0.025	0.004	9.83
Dilated cardiomyopathy	lvedvi	449027	923	0.203	0.033	8.94
Coronary_Artery_Disease	lvedv	449027	25195	-0.041	0.007	8.93
Dilated cardiomyopathy	lvedv	449027	923	0.198	0.033	8.64
Rheumatoid_arthritis	sv	449027	8751	-0.064	0.011	8.35
Pulmonary_embolism	svi	449027	5032	0.085	0.015	8.04
Mitral_valve_disease	lvef	449027	5978	-0.075	0.013	7.79
Myocardial_Infarction	lvedv	449027	19286	-0.042	0.008	7.57
Mitral_regurgitation	lvef	449027	3395	-0.097	0.018	7.34
Hypertension	lvesvi	449027	156957	0.018	0.003	7.11
Atrial_fibrillation_or_flutter	lvesv	449027	21367	-0.038	0.007	7.07
Hypothyroidism	lvedv	449027	28703	-0.032	0.006	6.77
Cardiac_surgery	sv	449027	8053	-0.056	0.011	6.13
Asthma	sv	449027	60959	0.022	0.004	6.01
Mitral_valve_disease	lvesvi	449027	5978	0.064	0.013	5.88
Atrial_fibrillation_or_flutter	lvesvi	449027	21367	-0.034	0.007	5.83
Mitral_regurgitation	lvesvi	449027	3395	0.082	0.017	5.65
Coronary_Artery_Disease	lvef	449027	25195	-0.031	0.007	5.46
Diabetes_Type_1	lvesv	449027	3914	-0.075	0.016	5.44
Hypertension	svi	449027	156957	-0.015	0.003	5.14
Breast_cancer	lvedvi	449027	16179	0.036	0.008	4.93
Myocardial_Infarction	lvef	449027	19286	-0.033	0.008	4.93
Diabetes_Type_2	lvedv	449027	28041	-0.027	0.006	4.82
Implantable_cardioverter_defibrillator	lvesvi	449027	1051	0.132	0.031	4.61
Coronary_Artery_Disease	lvedvi	449027	25195	-0.027	0.007	4.27
Peripheral_vascular_disease	lvesv	449027	6426	-0.051	0.013	4.25
Diabetes_Type_1	sv	449027	3914	-0.065	0.016	4.23
Hernia	lvedv	449027	59636	-0.018	0.004	4.20

Effect size, standard error, and P value are displayed for the association between manually curated phenotypes and the seven cardiac trait polygenic scores. Using Bonferroni correction to account for multiple testing (7 scores, 96 phenotypes), an association $P < 7.4 \times 10^{-5}$ with any of the 7 polygenic scores is considered significant and is listed in this table.

Supplementary Table 8: Characteristics of unrelated participants who did not undergo cardiac MRI

		**All Participants**
**N**		362922
**Sex**		
	Women	200744 (55 %)
	Men	162178 (45 %)
**Age at enrollment**		
	Mean (SD)	57 (8.1)

	Median [Q1-Q3]	58 [50 - 64]
**Ancestry**		
	African	2937 (1 %)
	East Asian	1303 (0 %)
	European	348778 (96 %)
	Other Ancestry	3670 (1 %)
	South Asian	6234 (2 %)
**BMI (kg/m^2)**		
	Mean (SD)	27 (4.8)
	Median [Q1-Q3]	27 [24 - 30]
	Missing	1572 (0.4%)
**Height (cm)**		
	Mean (SD)	170 (9.3)
	Median [Q1-Q3]	170 [160 - 180]
	Missing	1159 (0.3%)
**Weight (kg)**		
	Mean (SD)	78 (16)
	Median [Q1-Q3]	76 [66 - 87]
	Missing	1321 (0.4%)
**SBP (mmHg)**		
	Mean (SD)	140 (19)
	Median [Q1-Q3]	140 [120 - 150]
	Missing	446 (0.1%)
**DBP (mmHg)**		
	Mean (SD)	82 (10)
	Median [Q1-Q3]	82 [76 - 89]
	Missing	444 (0.1%)
**MET minutes/week**		
	Mean (SD)	2700 (3800)
	Median [Q1-Q3]	1500 [570 - 3200]
**Standard drinks/week**		
	Mean (SD)	12 (11)
	Median [Q1-Q3]	9.0 [5.3 - 16]
	Missing	118596 (32.7%)
**Pack year smoking history**		
	Mean (SD)	6.8 (15)
	Median [Q1-Q3]	0.0 [0.0 - 6.9]
**Smoking status at enrollment**		
	Current	39148 (11 %)
	Never	198743 (55 %)
	Prefer not to answer	1503 (0 %)
	Previous	123086 (34 %)
	unknown	442 (0 %)
**Diabetes diagnosis at baseline**		
	Absent	355468 (98 %)
	Present	7454 (2 %)
**Hypercholesterolemia diagnosis at baseline**		
	Absent	316299 (87 %)

	Present	46623 (13 %)
**Hypertension diagnosis at baseline**		
	Absent	261601 (72 %)
	Present	101321 (28 %)

This table presents the characteristics of the unrelated participants with genetic data who did not undergo cardiac MRI, and who were assessed for the ability of the polygenic scores to predict incident DCM.

Supplementary Table 9: Polygenic scores from cardiac MRI phenotypes are associated with incident DCM

Source of SNP Score	Hazard Ratio	95% CI Lower	95% CI Upper	P-value	SNPs
LVEDV	1.25	1.13	1.39	1.38E-05	22
LVEDVi	1.24	1.12	1.37	4.94E-05	14
LVEF	0.66	0.59	0.73	5.33E-15	22
LVESV	1.52	1.37	1.68	1.74E-15	32
**LVESVi**	**1.58**	**1.43**	**1.76**	**6.47E-18**	**28**
SV	1.12	1.01	1.24	3.08E-02	12
SVi	1.03	0.93	1.15	5.35E-01	8

“Hazard Ratio” represents the hazard ratio of a one standard deviation increase in the SNP score on the probability of developing dilated cardiomyopathy.

Supplementary Table 10: Characteristics of TTNtv carriers

		**Overall**
**N**		59
**Sex**		
	Women	34 (58 %)
	Men	25 (42 %)
**Ancestry**		
	Non-European	10 (17 %)
	European	49 (83 %)
**Age**		
	Mean (SD)	55 (7.9)
	Median [Q1-Q3]	56 [47 - 61]
**BMI (kg/m^2)**		
	Mean (SD)	27 (4.0)
	Median [Q1-Q3]	26 [24 - 30]
**Height (cm)**		
	Mean (SD)	170 (9.8)
	Median [Q1-Q3]	170 [160 - 180]
**Weight (kg)**		
	Mean (SD)	77 (13)
	Median [Q1-Q3]	75 [68 - 85]
**LVEDV (mL)**		
	Mean (SD)	140 (30)
	Median [Q1-Q3]	140 [120 - 160]

**LVESV (mL)**		
	Mean (SD)	58 (22)
	Median [Q1-Q3]	54 [43 - 69]
**LVEF (%)**		
	Mean (SD)	0.59 (0.080)
	Median [Q1-Q3]	0.61 [0.54 - 0.65]

Supplementary Table 11: Imputed TTNtv were not confirmed by exome sequencing

SNP	N Imputed	N Confirmed By Sequence	Cohort-wide MAF	INFO
rs565761937	40	0	8.21E-04	0.66
rs557312035	6	0	9.31E-05	0.45
rs565675340	6	0	8.26E-04	0.25
rs574660186	3	0	5.65E-05	0.95
rs548010682	2	0	4.35E-05	0.77
rs185589320	2	0	5.86E-05	0.86
rs112188483	2	0	3.45E-05	0.35
rs542074139	2	0	2.25E-05	0.77
rs140743001	1	0	1.62E-04	0.27
rs561946873	1	0	4.03E-05	0.40
rs145423907	1	0	6.13E-04	0.20

Genome-wide genotyping data was imputed and provided by the UK Biobank. Within the subset of 49,997 participants with exome sequencing data, 66 had imputed SNPs predicted to create a TTNtv. However, 0 of these 66 were confirmed in exome sequencing. Each imputed SNP, the number of participants imputed as having the SNP, the MAF of the SNP in the entire UK Biobank, and the imputation INFO score are displayed.

Supplementary Table 12: Clinical characteristics of European UK Biobank participants with cardiac MRI data

		Women	Men	All
**N**		17318	15437	32755
**Age at MRI**				
	Mean (SD)	64 (7.4)	65 (7.6)	64 (7.5)
	Median [Q1-Q3]	64 [58 - 69]	66 [59 - 71]	65 [58 - 70]
**Ancestry**				
	European	17318 (100 %)	15437 (100 %)	32755 (100 %)
**BMI (kg/m^2)**				
	Mean (SD)	26 (4.7)	27 (3.9)	26 (4.4)
	Median [Q1-Q3]	25 [23 - 28]	26 [24 - 29]	26 [23 - 29]
	Missing	497 (2.9%)	386 (2.5%)	883 (2.7%)
**Height (cm)**				

	Mean (SD)	160 (6.2)	180 (6.6)	170 (9.3)
	Median [Q1-Q3]	160 [160 - 170]	180 [170 - 180]	170 [160 - 180]
	Missing	450 (2.6%)	350 (2.3%)	800 (2.4%)
**Weight (kg)**				
	Mean (SD)	69 (13)	84 (13)	76 (15)
	Median [Q1-Q3]	67 [60 - 76]	82 [75 - 91]	75 [65 - 85]
	Missing	454 (2.6%)	372 (2.4%)	826 (2.5%)
**SBP (mmHg)**				
	Mean (SD)	130 (18)	140 (16)	140 (18)
	Median [Q1-Q3]	130 [120 - 140]	140 [130 - 150]	130 [120 - 150]
	Missing	7 (0.0%)	0 (0%)	7 (0.0%)
**DBP (mmHg)**				
	Mean (SD)	79 (9.7)	84 (9.6)	81 (9.9)
	Median [Q1-Q3]	79 [73 - 86]	84 [77 - 90]	81 [75 - 88]
	Missing	7 (0.0%)	0 (0%)	7 (0.0%)
**MET minutes/week**				
	Mean (SD)	2400 (3000)	2600 (3400)	2500 (3200)
	Median [Q1-Q3]	1500 [620 - 2900]	1600 [690 - 3200]	1500 [660 - 3100]
**Standard drinks/week**				
	Mean (SD)	9.2 (6.9)	15 (11)	12 (9.4)
	Median [Q1-Q3]	7.3 [4.5 - 12]	12 [7.3 - 19]	9.3 [6.0 - 16]
	Missing	4978 (28.7%)	2563 (16.6%)	7541 (23.0%)
**Pack year smoking history**				
	Mean (SD)	3.6 (9.0)	5.7 (13)	4.6 (11)
	Median [Q1-Q3]	0.0 [0.0 - 0.0]	0.0 [0.0 - 3.8]	0.0 [0.0 - 0.0]
**Smoking status at enrollment**				
	Current	861 (5 %)	1090 (7 %)	1951 (6 %)
	Never	11136 (64 %)	8847 (57 %)	19983 (61 %)
	Prefer_not_to_answer	34 (0 %)	30 (0 %)	64 (0 %)
	Previous	5287 (31 %)	5470 (35 %)	10757 (33 %)
**LVEDV**				
	Mean (SD)	120 (20)	150 (28)	140 (29)
	Median [Q1-Q3]	120 [110 - 140]	150 [130 - 170]	130 [120 - 150]
**LVEDVi**				
	Mean (SD)	70 (11)	76 (14)	73 (12)
	Median [Q1-Q3]	70 [63 - 77]	76 [67 - 85]	72 [65 - 80]
	Missing	480 (2.8%)	381 (2.5%)	861 (2.6%)
**LVESV**				
	Mean (SD)	42 (11)	58 (17)	49 (16)
	Median [Q1-Q3]	41 [34 - 48]	56 [47 - 67]	47 [38 - 58]
**LVESVi**				
	Mean (SD)	24 (6.1)	29 (8.0)	26 (7.5)
	Median [Q1-Q3]	23 [20 - 27]	28 [24 - 33]	25 [21 - 30]
	Missing	480 (2.8%)	381 (2.5%)	861 (2.6%)
**LVEF**				

	Mean (SD)	0.67 (0.052)	0.63 (0.058)	0.65 (0.058)
	Median [Q1-Q3]	0.67 [0.64 - 0.70]	0.63 [0.59 - 0.66]	0.65 [0.61 - 0.69]
**SV**				
	Mean (SD)	81 (12)	96 (17)	88 (16)
	Median [Q1-Q3]	81 [73 - 89]	95 [85 - 110]	87 [77 - 98]
**SVi**				
	Mean (SD)	46 (6.6)	47 (8.1)	47 (7.4)
	Median [Q1-Q3]	46 [42 - 51]	47 [42 - 53]	47 [42 - 52]
	Missing	480 (2.8%)	381 (2.5%)	861 (2.6%)

Supplementary Table 13: Heritability in the full sample and in the European-specific subset

	**Heritability**	
	**All participants**	**European participants**
**LVEDV**	0.426	0.445
**LVESV**	0.400	0.417
**SV**	0.342	0.364
**LVEF**	0.313	0.332

SNP heritability was assessed with BOLT-REML. A sensitivity analysis that excluded all outliers from the genetically European subset revealed similar heritability estimates to those from the main analyses.

Supplementary Table 14: LD score regression in the European-specific analysis

	**Lambda**	**Intercept**
**LVEDV**	1.1459	1.0258
**LVEDVi**	1.1459	1.0047
**LVESV**	1.1459	1.0088
**LVESVi**	1.1459	0.9973
**LVEF**	1.0957	0.9901
**SV**	1.1459	1.0277
**SVi**	1.0957	1.0092

Lambda represents the genomic inflation factor. Intercept represents the intercept from LD score regression.

Supplementary Table 15: Loci significant at various P value thresholds in the European-specific analysis

P threshold	LVEDV	LVEDVi	LVESV	LVESVi	LVEF	SV	SVi	Total distinct loci
5.00E-08	19	10	19	28	21	11	7	49
1.00E-08	15	5	16	16	15	6	4	28
5.00E-09	13	5	14	15	15	5	3	26
1.00E-09	10	5	11	14	12	4	2	19

Under each trait, the number of loci significant at the P value threshold from the leftmost column is displayed. The *Total distinct loci* column indicates the total number of distinct loci across all phenotypes, counting loci found for more than one trait just once.

Supplementary Table 16: Loci newly achieving genome-wide significance in the European-specific analysis

Trait	SNP	CHR	BP	A1	A0	A1 Freq	A1 Freq Euro	INFO	BETA	BETA Euro	P	P Euro	Nearest Gene	Novel in Study
lvedv	rs71517989	8	56877592	A	AT	0.554	0.554	0.99	0.0314	0.0367	1.60E-07	7.70E-09	LYN	Yes
lvef	rs36234	16	2201270	G	C	0.637	0.635	0.91	0.0399	0.0426	6.90E-08	3.00E-08	RAB26	No
lvesv	rs113781447	19	2317945	T	A	0.982	0.982	0.92	-0.1315	-0.1419	1.20E-07	3.20E-08	LSM7	Yes
lvesv	rs377751939	5	138734734	T	C	0.807	0.814	0.84	0.0456	0.0495	6.00E-08	2.50E-08	SPATA24	No
sv	rs149613324	11	104215908	G	C	0.991	0.991	0.84	-0.1827	-0.2070	2.80E-07	2.40E-08	PDGFD	Yes
svi	rs10258757	7	98970675	C	T	0.863	0.872	1.00	-0.0586	-0.0663	7.70E-08	7.70E-09	ARPC1A	Yes

BP is the base pair distance, keyed to GRCh37. A1 is the effect allele, A0 is the non-effect allele. A1 Freq Euro represents the effect allele frequency in the European-specific analysis. BETA Euro and P Euro, respectively, represent the effect size and P value in the European-specific analysis. Novel in Study indicates whether a locus was detected for another trait in the main analysis (*No*) or whether the locus was not detected in the main analysis (*Yes*).

Supplementary Table 17: Concordance of effect estimates from lead SNPs in the main analysis and the European-specific analysis

Trait	P Threshold	Evaluated SNPs	R^2
lvedv	1	14457773	0.52
	0.05	910097	0.91
	0.005	130905	0.96
	0.0005	26359	0.99
	5.00E-05	8183	0.99
	5.00E-06	3414	1.00
	5.00E-07	1869	1.00
	5.00E-08	1150	1.00
	5.00E-09	552	1.00
lvedvi	1	14459489	0.52
	0.05	867566	0.91
	0.005	115475	0.97
	0.0005	19745	0.99
	5.00E-05	5466	1.00
	5.00E-06	1690	1.00
	5.00E-07	832	1.00
	5.00E-08	513	1.00
	5.00E-09	256	1.00
lvesv	1	14458151	0.52
	0.05	907979	0.89
	0.005	134774	0.96
	0.0005	28470	0.99
	5.00E-05	9758	1.00
	5.00E-06	4459	1.00
	5.00E-07	2743	1.00
	5.00E-08	1788	1.00
	5.00E-09	1159	1.00
lvesvi	1	14459698	0.52
	0.05	883056	0.89
	0.005	122307	0.95
	0.0005	23158	0.99

	5.00E-05	7774	0.99
	5.00E-06	3434	1.00
	5.00E-07	2322	1.00
	5.00E-08	1728	1.00
	5.00E-09	1292	1.00
lvef	1	14457483	0.51
	0.05	859758	0.88
	0.005	119329	0.95
	0.0005	24647	0.98
	5.00E-05	7924	1.00
	5.00E-06	4284	1.00
	5.00E-07	2266	1.00
	5.00E-08	1630	1.00
	5.00E-09	1166	1.00
sv	1	14457631	0.52
	0.05	859053	0.92
	0.005	115812	0.97
	0.0005	21115	0.99
	5.00E-05	4696	0.99
	5.00E-06	1754	1.00
	5.00E-07	755	1.00
	5.00E-08	400	1.00
	5.00E-09	216	1.00
svi	1	14458828	0.52
	0.05	828266	0.91
	0.005	105162	0.96
	0.0005	15729	0.99
	5.00E-05	3262	0.99
	5.00E-06	844	1.00
	5.00E-07	285	1.00
	5.00E-08	91	1.00
	5.00E-09	8	1.00

For each trait, effect estimates (Beta) for each SNP in the main analysis were compared with those in the European-specific analysis in a linear model. Using different subsets of SNPs based on the P value in the main analysis, agreement was assessed by the overall model fit (r^2).

Supplementary Table 18: Directional concordance of genome-wide significant SNPs from the main analysis and the European-specific analysis

Trait	Evaluated SNPs	SNPs at P < 5E-08 in the main analysis	Sign mismatches in the European-specific analysis
lvedv	14528119	1150	0
lvedvi	14529827	513	0
lvesv	14528119	1788	0
lvesvi	14529827	1728	0
lvef	14528119	1630	0
sv	14528119	400	0
svi	14529827	91	0

No lead SNPs with P < 5e-8 in the main analysis had an opposite direction of effect in the European-specific analysis.

Supplementary Table 19: Characteristics of unrelated European participants who did not undergo cardiac MRI

		All European Participants
**N**		313885
**Sex**		
	Women	173092 (55 %)
	Men	140793 (45 %)
**Age at enrollment**		
	Mean (SD)	57 (8.0)
	Median [Q1-Q3]	59 [51 - 64]
**Ancestry**		
	European	313885 (100 %)
**BMI (kg/m^2)**		
	Mean (SD)	27 (4.8)
	Median [Q1-Q3]	27 [24 - 30]
	Missing	1062 (0.3%)
**Height (cm)**		
	Mean (SD)	170 (9.3)
	Median [Q1-Q3]	170 [160 - 180]
	Missing	717 (0.2%)
**Weight (kg)**		
	Mean (SD)	78 (16)
	Median [Q1-Q3]	77 [67 - 88]
	Missing	945 (0.3%)
**SBP (mmHg)**		

	Mean (SD)	140 (19)
	Median [Q1-Q3]	140 [130 - 150]
	Missing	310 (0.1%)
**DBP (mmHg)**		
	Mean (SD)	82 (10)
	Median [Q1-Q3]	82 [76 - 89]
	Missing	308 (0.1%)
**MET minutes/week**		
	Mean (SD)	2700 (3800)
	Median [Q1-Q3]	1500 [580 - 3300]
**Standard drinks/week**		
	Mean (SD)	12 (11)
	Median [Q1-Q3]	9.3 [5.5 - 16]
	Missing	93280 (29.7%)
**Pack year smoking history**		
	Mean (SD)	7.1 (15)
	Median [Q1-Q3]	0.0 [0.0 - 7.5]
**Smoking status at enrollment**		
	Current	33006 (11 %)
	Never	170406 (54 %)
	Prefer not to answer	1108 (0 %)
	Previous	109365 (35 %)
**Diabetes diagnosis at baseline**		
	Absent	307966 (98 %)
	Present	5919 (2 %)
**Hypercholesterolemia diagnosis at baseline**		
	Absent	273836 (87 %)
	Present	40049 (13 %)
**Hypertension diagnosis at baseline**		
	Absent	226196 (72 %)
	Present	87689 (28 %)

This table presents the characteristics of the unrelated participants in the European genetic subset who did not undergo cardiac MRI, and who were assessed for the ability of the polygenic scores to predict incident DCM.

Supplementary Table 20: Polygenic scores from cardiac MRI phenotypes are associated with incident DCM in the European-specific analysis

Source of SNP Score	Hazard Ratio	95% CI Lower	95% CI Upper	P-value

lvedv	1.32	1.18	1.48	2.23E-06
lvedvi	1.23	1.10	1.38	1.91E-04
lvef	0.65	0.59	0.73	1.52E-13
lvesv	1.48	1.32	1.65	7.77E-12
**lvesvi**	**1.56**	**1.40**	**1.75**	**9.10E-15**
svi	1.17	1.05	1.30	4.82E-03
svi	1.01	0.91	1.13	8.44E-01

“Hazard Ratio” represents the hazard ratio of a one standard deviation increase in the SNP score on the probability of developing dilated cardiomyopathy.

Supplementary Figures

Supplementary Figure 1: Flow diagram for UK Biobank GWAS participants

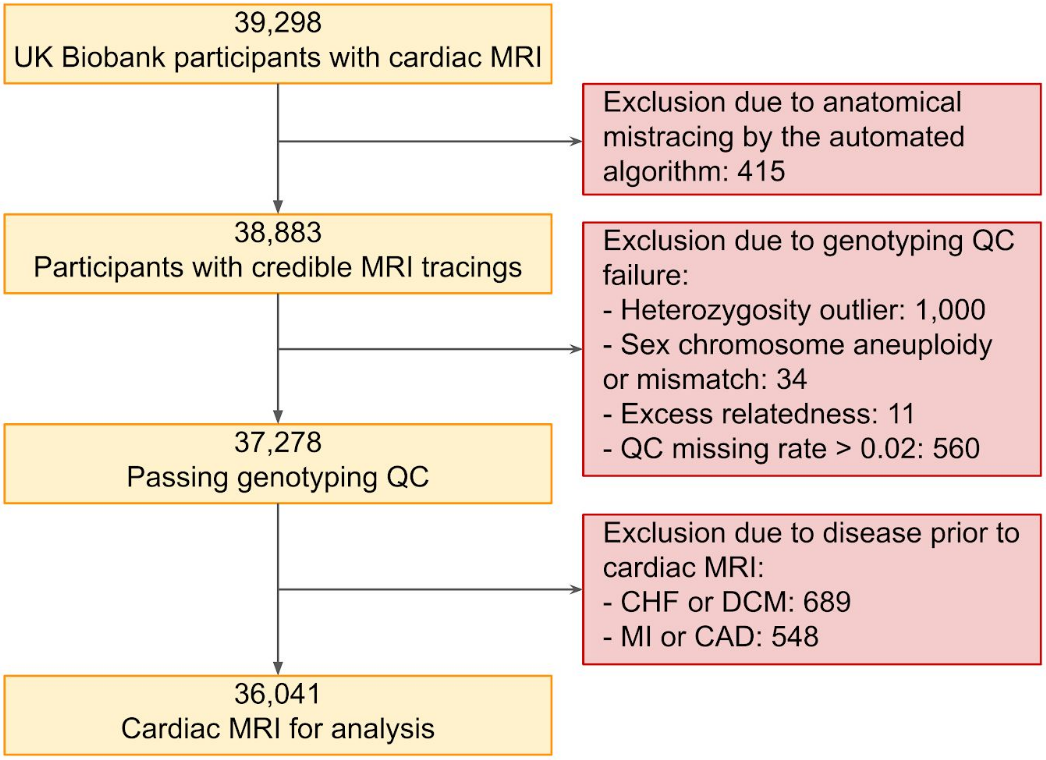

Supplementary Figure 2: Sex-stratified distributions of cardiac MRI phenotypes

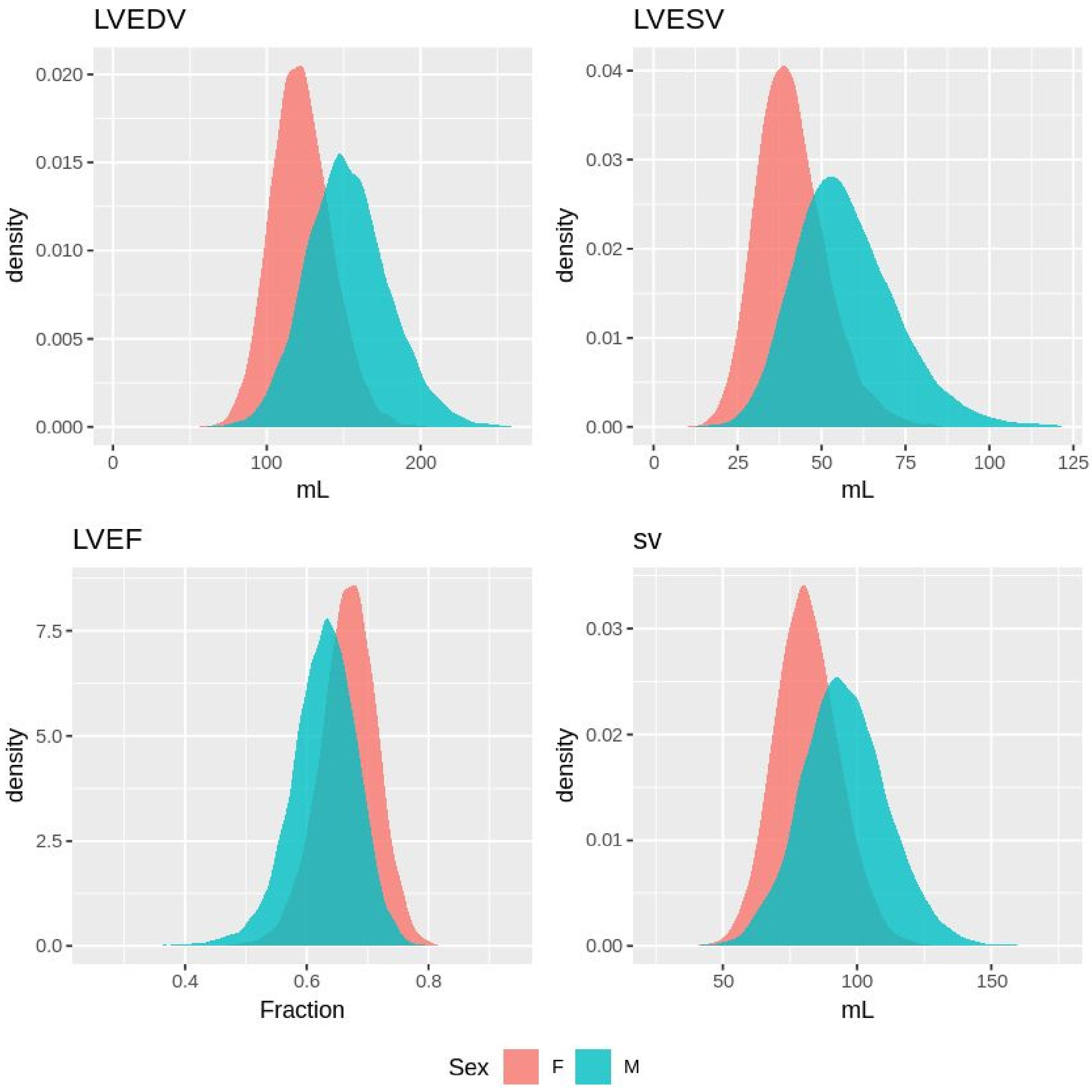

The population distributions of each of the non-BSA-indexed cardiac MRI phenotypes are represented via density plots.

Supplementary Figure 3: Cross-trait correlation

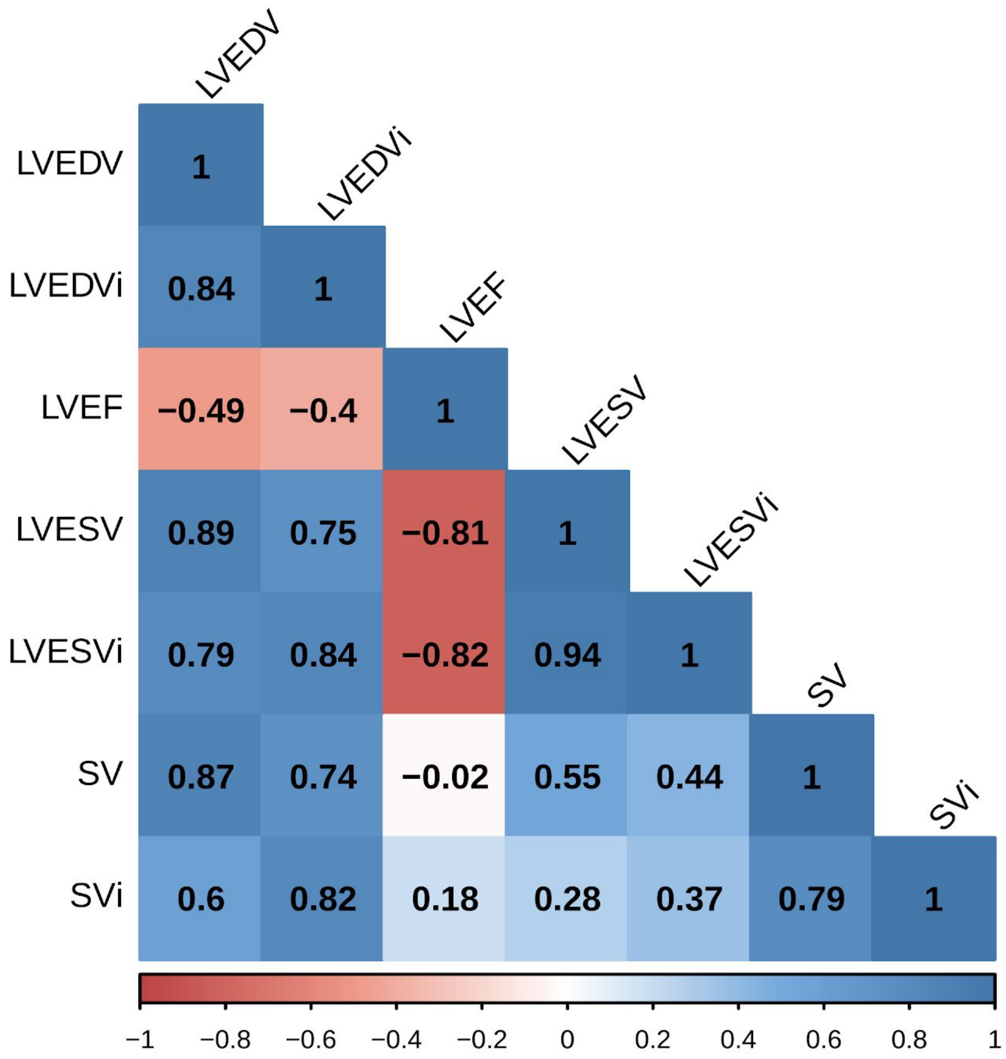

For each cardiac MRI phenotype pair, the Pearson correlation is represented by a number ranging from -1 (perfect anticorrelation) to 1 (perfect correlation), representing the degree to which each trait is correlated with another.

Supplementary Figure 4: Genetic correlation

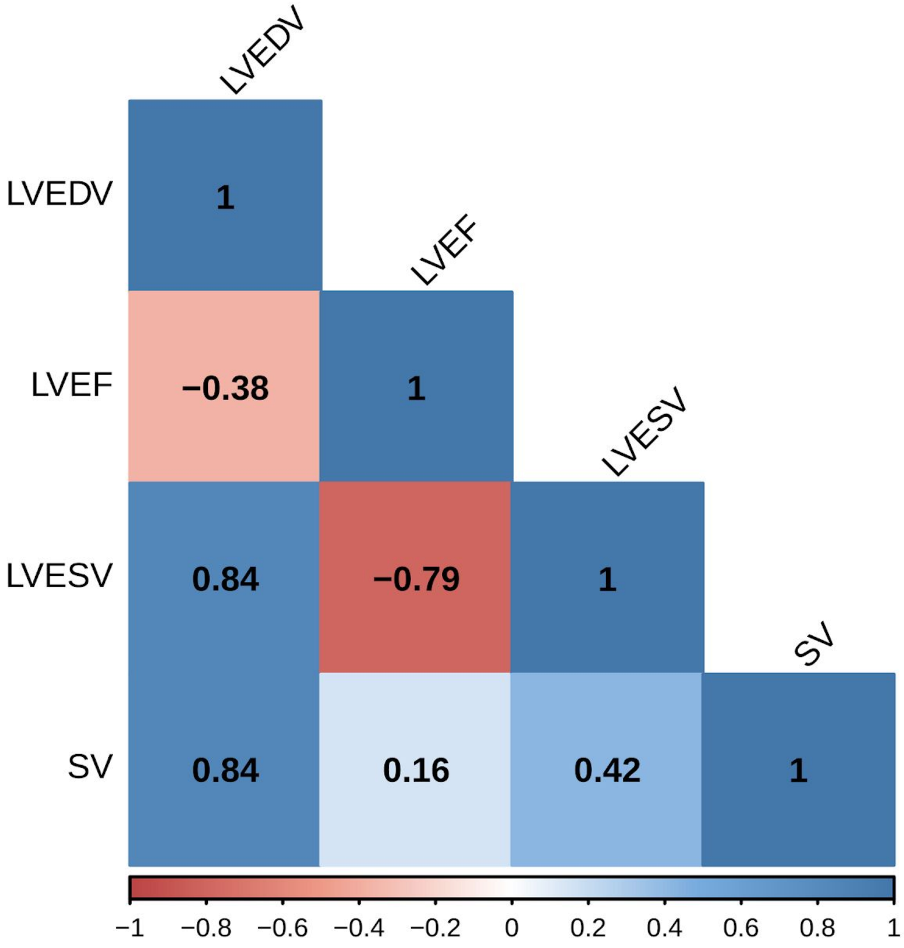

For each of the raw cardiac MRI phenotype pairs (excluding body surface area-indexed traits), the genetic correlation as measured by BOLT REML is represented by a number ranging from -1 (perfect anticorrelation) to 1 (perfect correlation).

Supplementary Figure 5: MAGMA gene expression enrichment

For each cardiac phenotype, the associations of gene expression sets from GTEx were evaluated using MAGMA, running on the FUMA platform v1.3.4b. Gene expression sets significantly enriched after Bonferroni correction are highlighted in red.

LVEDV

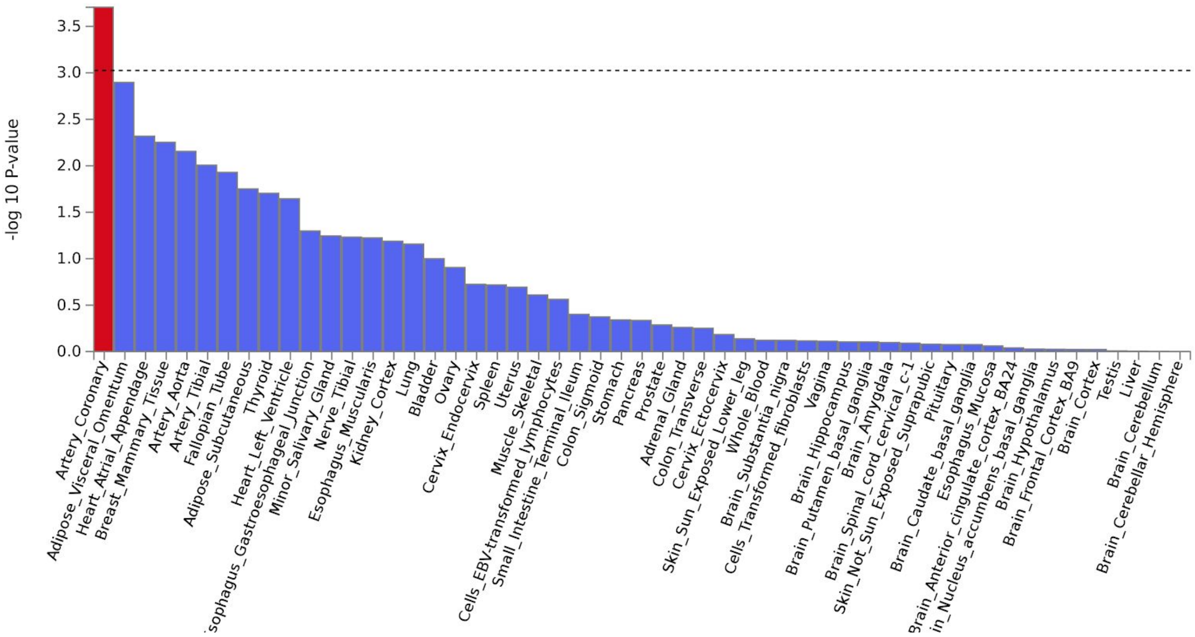

LVESV

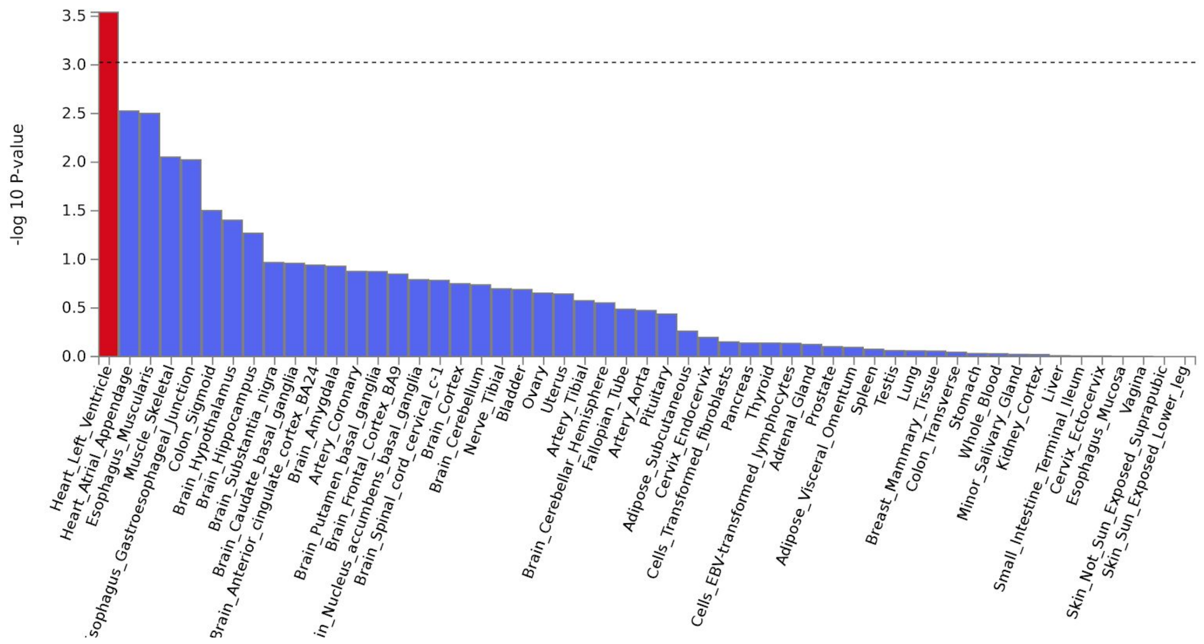

LVEF

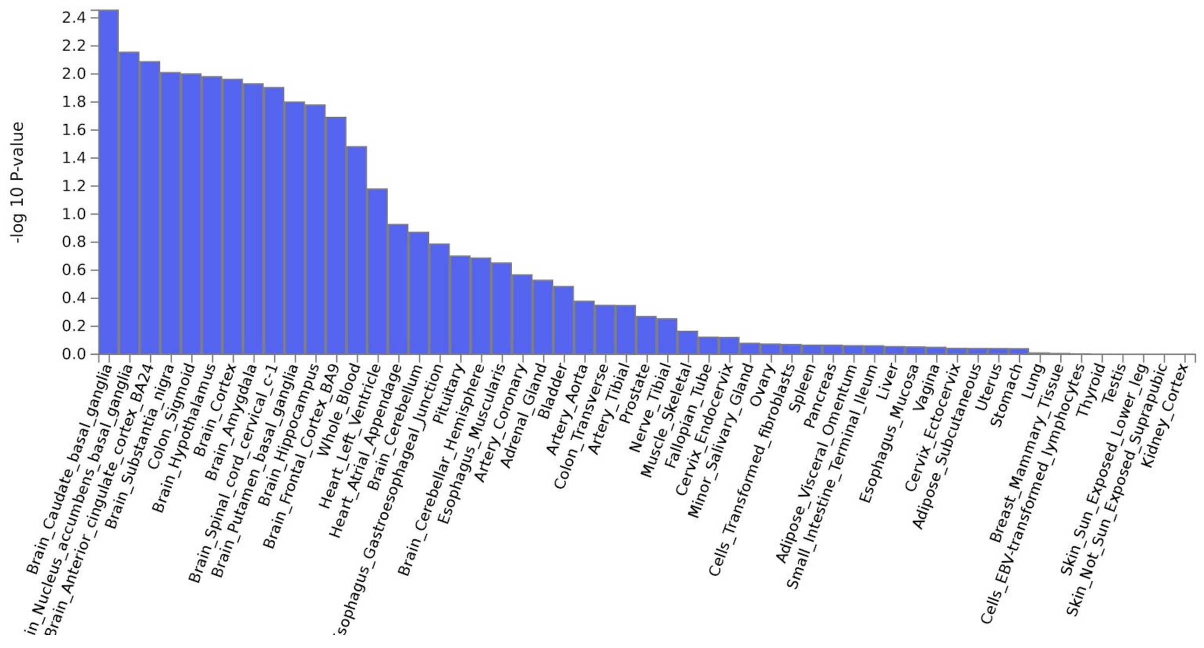

SV

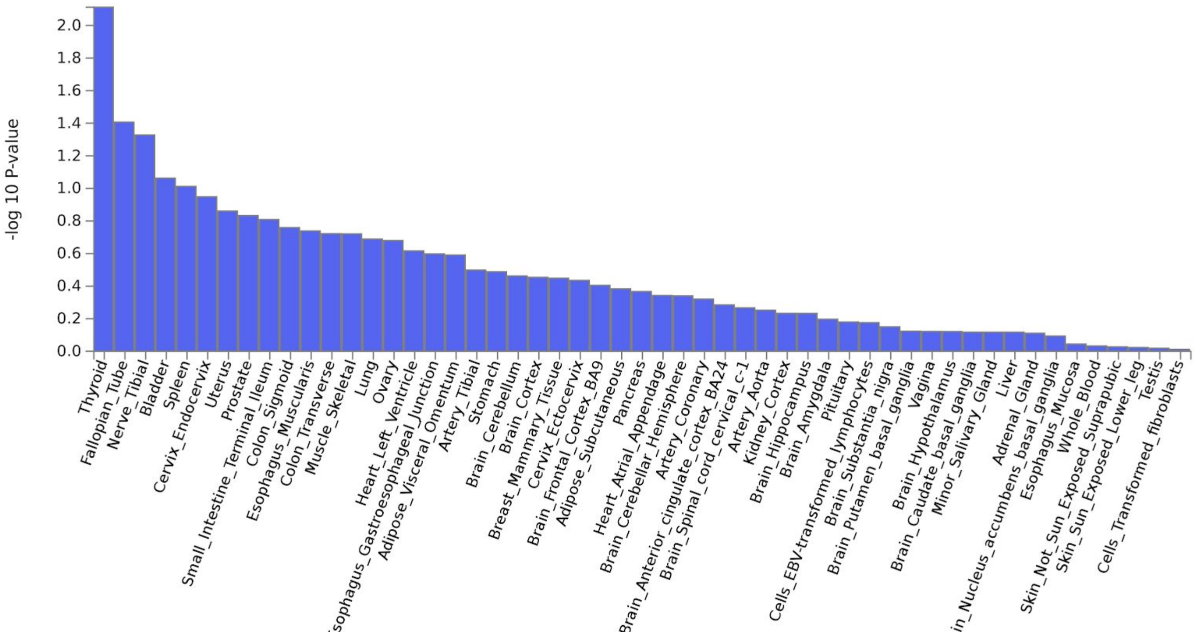
-log 10 P-value
0.0
0.2
0.4
0.6
0.8
1.0
1.2
1.4
1.6
1.8
2.0
Thyroid
Fallopian_Tube
Nerve_Tibial
Bladder
Spleen
Cervix_Endocervix
Uterus
Prostate
Small_Intestine_Terminal_Ileum
Colon_Sigmoid
Esophagus_Muscularis
Colon_Transverse
Muscle_Skeletal
Lung
Ovary
Heart_Left_Ventricle
sophagus_Gastroesophageal_Junction
Adipose_Visceral_Omentum
Artery_Tibial
Stomach
Brain_Cerebellum
Brain_Cortex
Breast_Mammary_Tissue
Cervix_Ectocervix
Brain_Frontal_Cortex_BA9
Adipose_Subcutaneous
Pancreas
Heart_Atrial_Appendage
Brain_Cerebellar_Hemisphere
Artery_Coronary
Brain_Anterior_cingulate_cortex_BA24
Brain_Spinal_cord_cervical_c-1
Artery_Aorta
Kidney_Cortex
Brain_Hippocampus
Brain_Amygdala
Pituitary
Cells_EBV-transformed_lymphocytes
Brain_Substantia_nigra
Brain_Putamen_basal_ganglia
Vagina
Brain_Hypothalamus
Brain_Caudate_basal_ganglia
Minor_Salivary_Gland
Liver
Adrenal_Gland
in_Nucleus_accumbens_basal_ganglia
Esophagus_Mucosa
Whole_Blood
Skin_Not_Sun_Exposed_Suprapubic
Skin_Sun_Exposed_Lower_leg
Testis
Cells_Transformed_fibroblasts

LVEDVi

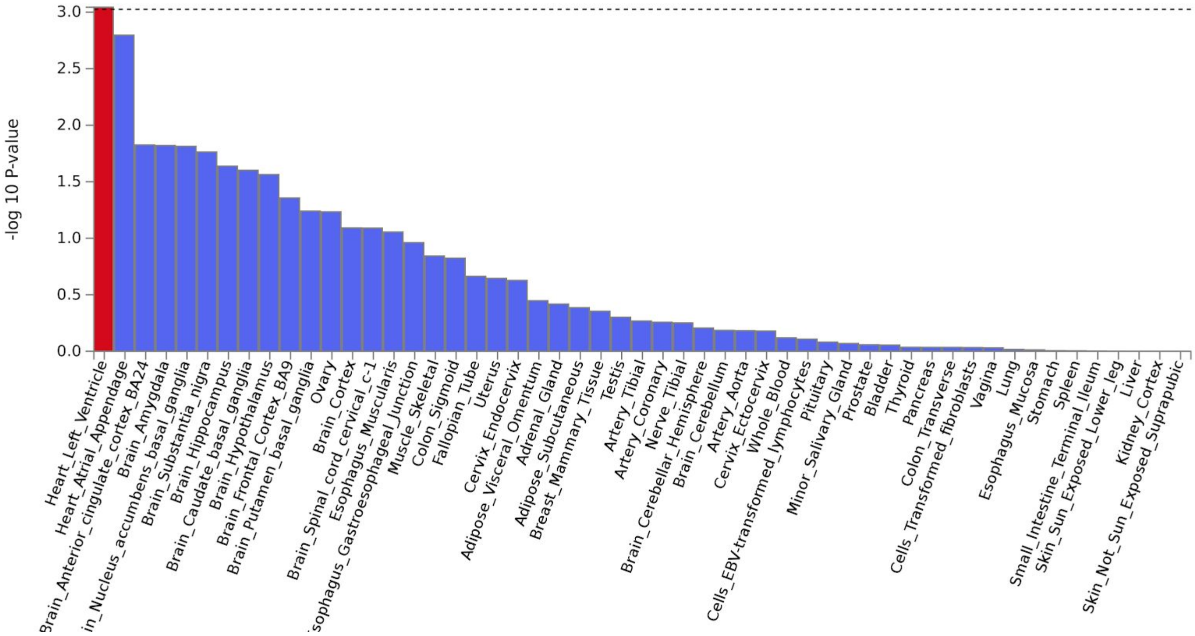
-log 10 P-value
0.0
0.5
1.0
1.5
2.0
2.5
3.0
Heart_Left_Ventricle
Heart_Atrial_Appendage
Brain_Anterior_cingulate_cortex_BA24
Brain_Amygdala
in_Nucleus_accumbens_basal_ganglia
Brain_Substantia_nigra
Brain_Hippocampus
Brain_Caudate_basal_ganglia
Brain_Hypothalamus
Brain_Frontal_Cortex_BA9
Brain_Putamen_basal_ganglia
Ovary
Brain_Cortex
Brain_Spinal_cord_cervical_c-1
Esophagus_Muscularis
sophagus_Gastroesophageal_Junction
Muscle_Skeletal
Colon_Sigmoid
Fallopian_Tube
Uterus
Cervix_Endocervix
Adipose_Visceral_Omentum
Adrenal_Gland
Adipose_Subcutaneous
Breast_Mammary_Tissue
Testis
Artery_Tibial
Artery_Coronary
Nerve_Tibial
Brain_Cerebellar_Hemisphere
Brain_Cerebellum
Artery_Aorta
Cervix_Ectocervix
Whole_Blood
Cells_EBV-transformed_lymphocytes
Pituitary
Minor_Salivary_Gland
Prostate
Bladder
Thyroid
Pancreas
Colon_Transverse
Cells_Transformed_fibroblasts
Vagina
Lung
Esophagus_Mucosa
Stomach
Spleen
Small_Intestine_Terminal_Ileum
Skin_Sun_Exposed_Lower_leg
Liver
Kidney_Cortex
Skin_Not_Sun_Exposed_Suprapubic

LVESVi

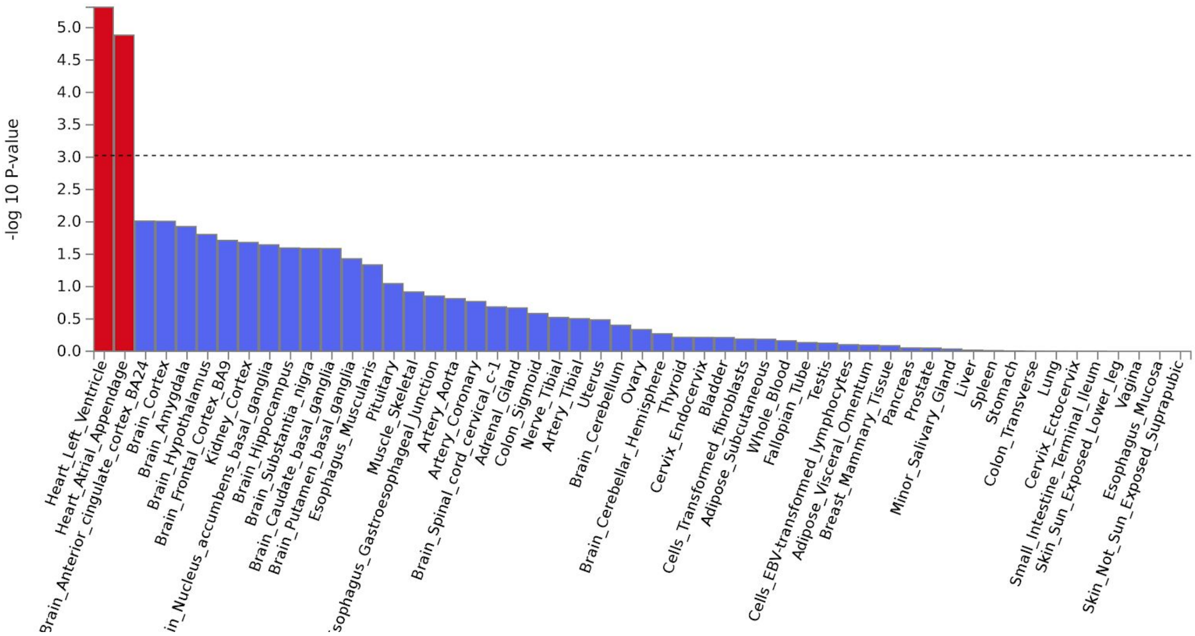
-log 10 P-value
5.0
4.5
4.0
3.5
3.0
2.5
2.0
1.5
1.0
0.5
0.0
Heart_Left_Ventricle
Heart_Atrial_Appendage
rain_Anterior_cingulate_cortex_BA24
Brain_Cortex
Brain_Amygdala
Brain_Hypothalamus
Brain_Frontal_Cortex_BA9
Kidney_Cortex
in_Nucleus_accumbens_basal_ganglia
Brain_Hippocampus
Brain_Substantia_nigra
Brain_Caudate_basal_ganglia
Brain_Putamen_basal_ganglia
Esophagus_Muscularis
Pituitary
Muscle_Skeletal
sophagus_Gastroesophageal_Junction
Artery_Aorta
Artery_Coronary
Brain_Spinal_cord_cervical_c-1
Adrenal_Gland
Colon_Sigmoid
Nerve_Tibial
Artery_Tibial
Uterus
Brain_Cerebellum
Ovary
Brain_Cerebellar_Hemisphere
Thyroid
Cervix_Endocervix
Bladder
Cells_Transformed_fibroblasts
Adipose_Subcutaneous
Whole_Blood
Fallopian_Tube
Testis
Cells_EBV-transformed_lymphocytes
Adipose_Visceral_Omentum
Breast_Mammary_Tissue
Pancreas
Prostate
Minor_Salivary_Gland
Liver
Spleen
Stomach
Colon_Transverse
Lung
Cervix_Ectocervix
Small_Intestine_Terminal_Ileum
Skin_Sun_Exposed_Lower_leg
Vagina
Esophagus_Mucosa
Skin_Not_Sun_Exposed_Suprapubic

SVi

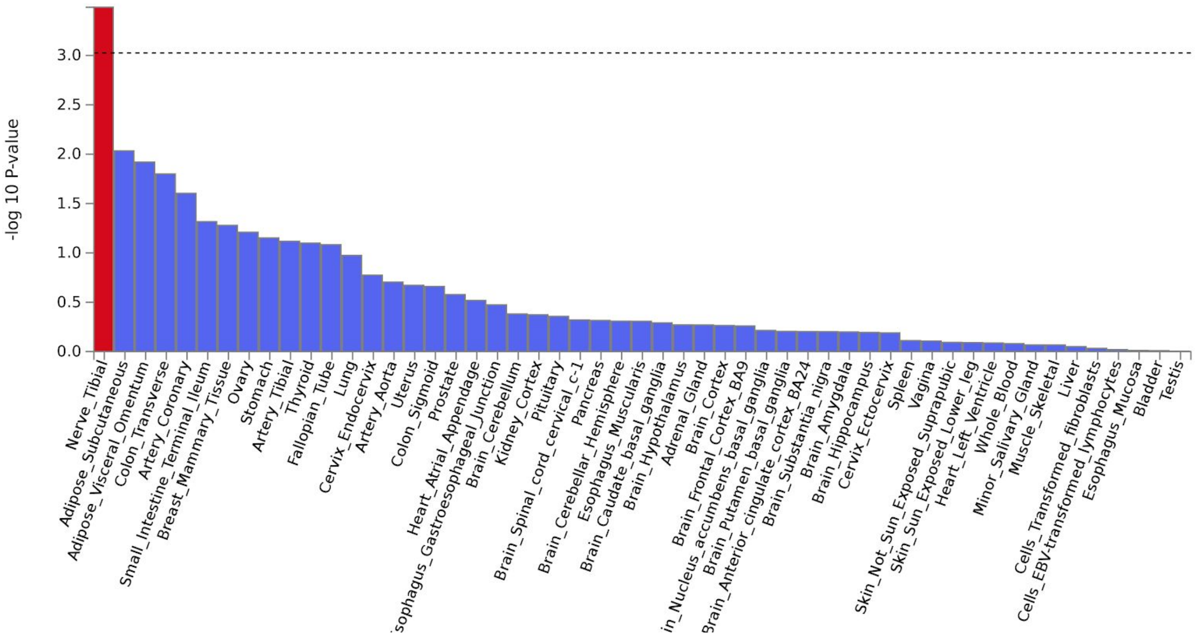
-log 10 P-value
3.0
2.5
2.0
1.5
1.0
0.5
0.0
Nerve_Tibial
Adipose_Subcutaneous
Adipose_Visceral_Omentum
Colon_Transverse
Artery_Coronary
Small_Intestine_Terminal_Ileum
Breast_Mammary_Tissue
Ovary
Stomach
Artery_Tibial
Thyroid
Fallopian_Tube
Lung
Cervix_Endocervix
Artery_Aorta
Uterus
Colon_Sigmoid
Prostate
Heart_Atrial_Appendage
sophagus_Gastroesophageal_Junction
Brain_Cerebellum
Kidney_Cortex
Pituitary
Brain_Spinal_cord_cervical_c-1
Pancreas
Brain_Cerebellar_Hemisphere
Esophagus_Muscularis
Brain_Caudate_basal_ganglia
Brain_Hypothalamus
Adrenal_Gland
Brain_Cortex
Brain_Frontal_Cortex_BA9
in_Nucleus_accumbens_basal_ganglia
Brain_Putamen_basal_ganglia
rain_Anterior_cingulate_cortex_BA24
Brain_Substantia_nigra
Brain_Amygdala
Brain_Hippocampus
Cervix_Ectocervix
Spleen
Vagina
Skin_Not_Sun_Exposed_Suprapubic
Skin_Sun_Exposed_Lower_leg
Heart_Left_Ventricle
Whole_Blood
Minor_Salivary_Gland
Muscle_Skeletal
Liver
Cells_Transformed_fibroblasts
Cells_EBV-transformed_lymphocytes
Esophagus_Mucosa
Bladder
Testis

Supplementary Figure 6: GWAS loci are found nearby to more cardiomyopathy-related genes than expected by chance

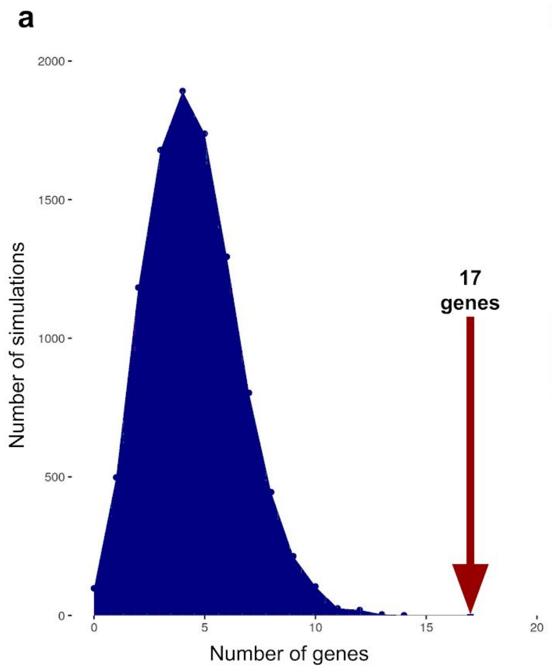

a. In 10,000 simulations of SNPs matched to our GWAS lead SNPs in their statistical properties, on average 2 Mendelian cardiomyopathy genes were located within 500kb of those SNPs (range: 0-11 genes). In contrast, 17 Mendelian genes were found within 500kb of the actual SNPs from our study (dotted red vertical line; one-tailed permutation P = 1×10^{-4}). **b.** Mendelian cardiomyopathy-related genes from **Supplementary Table 5** found within 500kb of genome-wide significant SNPs associated with cardiomyopathy and cardiac MRI phenotypes are plotted in a Venn diagram. Genes found only near loci discovered in this GWAS of cardiac

MRI phenotypes are located in the pink circle (12 genes); those found only in prior cardiomyopathy GWAS appear in the yellow circle (0 genes); and those found in both are in the overlapping orange area (5 genes).

Supplementary Figure 7: PheWAS

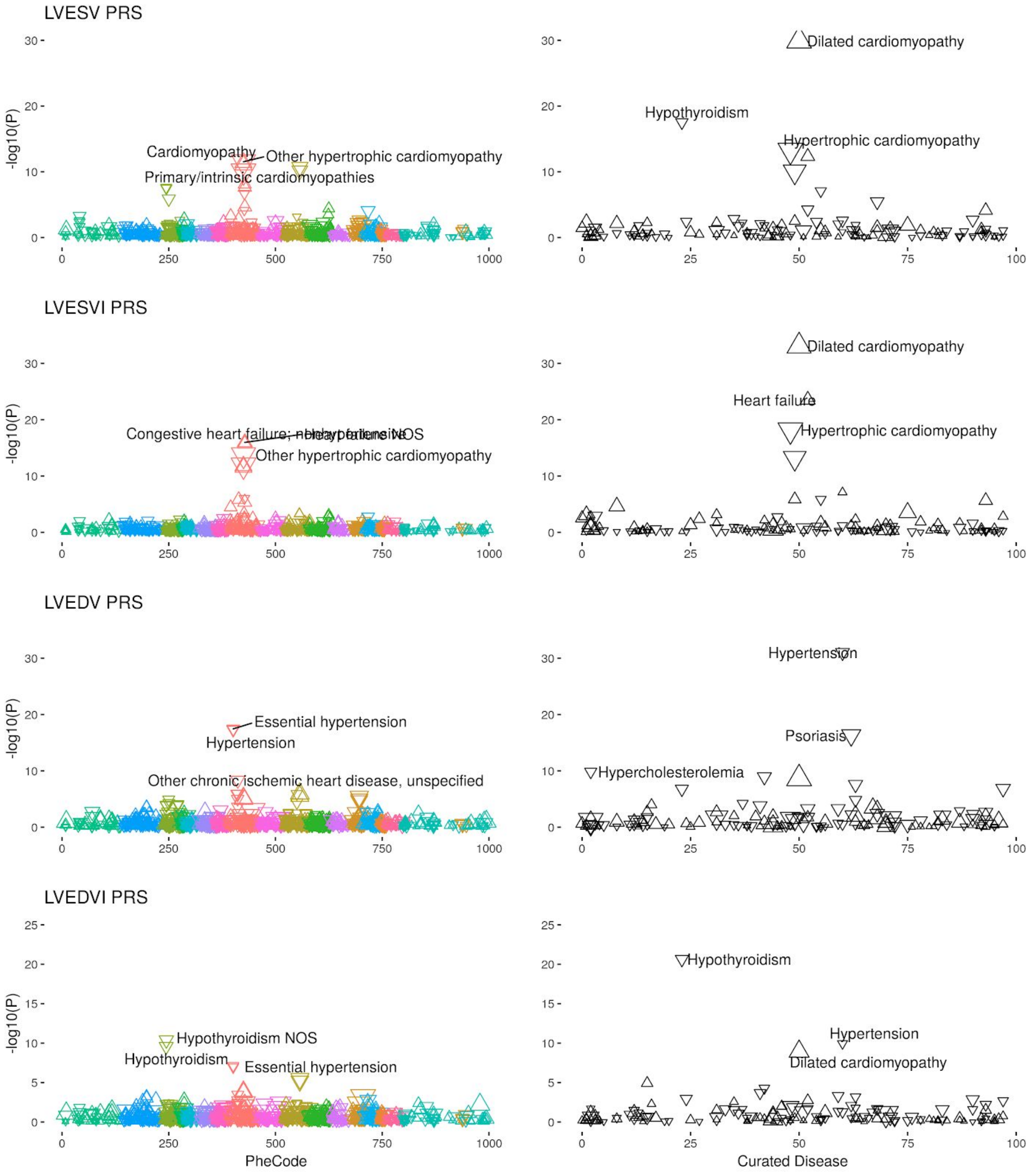

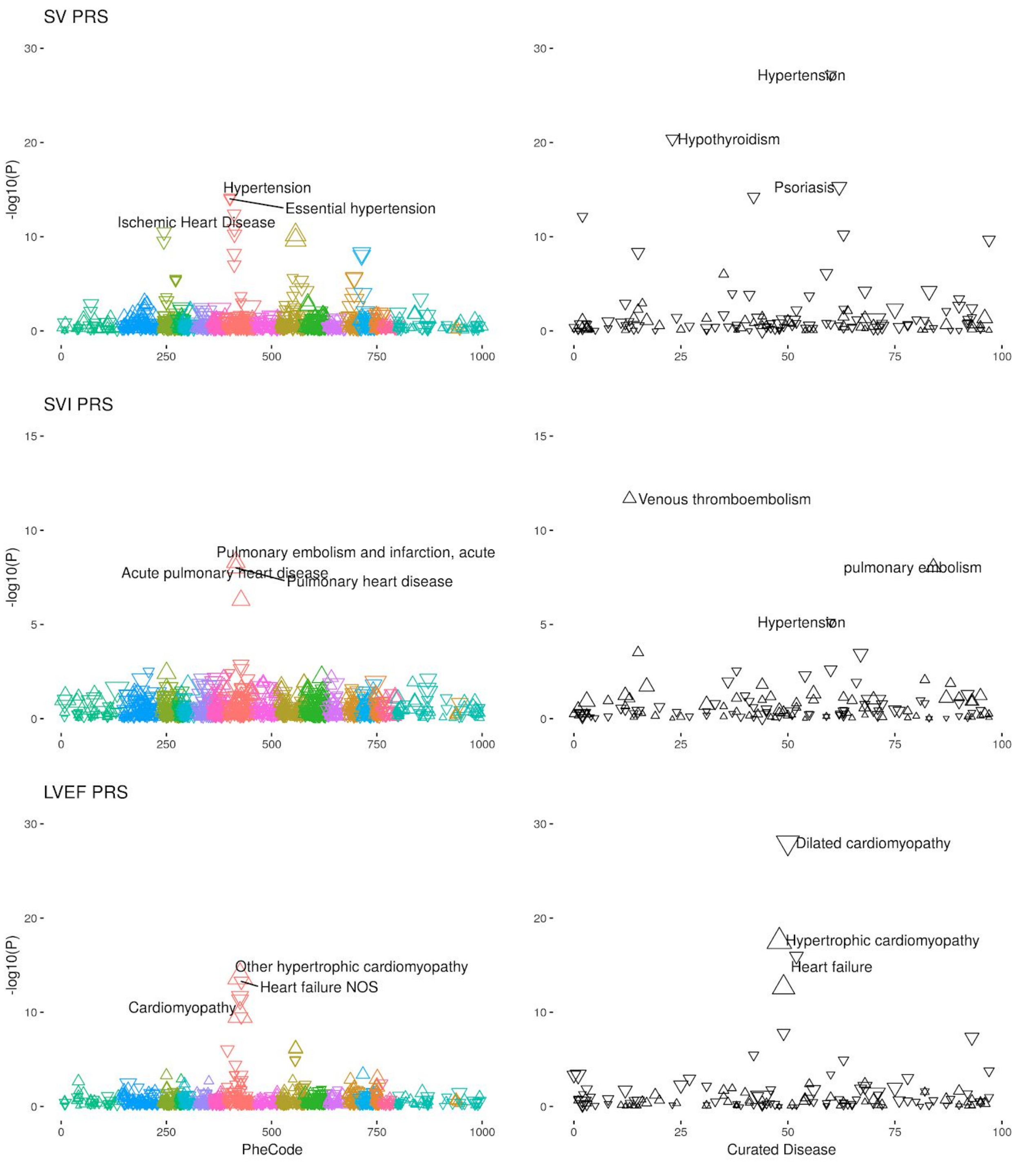

For each of the 7 traits for which a genome-wide association study was performed, the polygenic score was applied to two sets of phenotypes. On the left, colored by PheCode grouping, are PheCode-defined phenotypes. On the right in black-and-white are the curated

disease phenotypes defined in **Supplementary Table 1**. The X-axis is manually defined and arbitrary, though attempts to cluster similar phenotypes. The Y-axis represents the -log10 of the P-value of association between the polygenic score and the phenotype in a logistic regression model adjusted for age at enrollment, the genotyping array, sex, and the first five principal components of ancestry. The three most strongly associated phenotypes for each group are labeled.

Supplementary Figure 8: SNP effect estimates in the main analysis and in the European-specific analysis

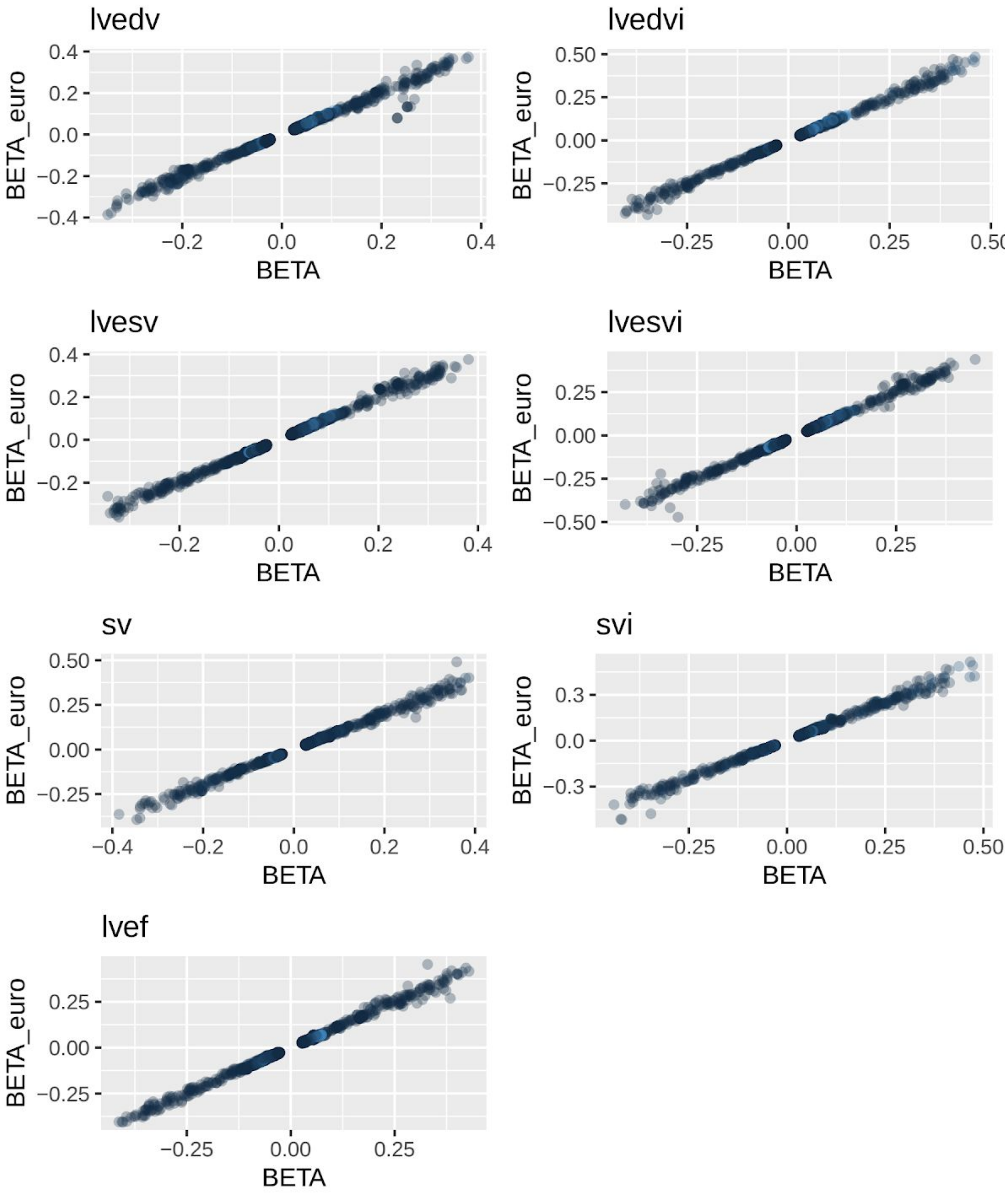

Each point represents one SNP; the X-coordinate represents the SNP's effect estimate in the main analysis (BETA) and the Y-coordinate represents its effect estimate in the European-specific analysis (BETA_euro). Perfect correlation would be reflected in a line of y = x. All SNPs with $P < 5\times10^{-5}$ for each trait are plotted.

Supplementary References

1. Roselli, C. *et al.* Multi-Ethnic Genome-wide Association Study for Atrial Fibrillation. *Nat. Genet.* **50**, 1225–1233 (2018).
2. Bycroft, C. *et al.* The UK Biobank resource with deep phenotyping and genomic data. *Nature* **562**, 203 (2018).
3. Bild, D. E. *et al.* Multi-Ethnic Study of Atherosclerosis: objectives and design. *Am. J. Epidemiol.* **156**, 871–881 (2002).
4. Natarajan, P. *et al.* Deep-coverage whole genome sequences and blood lipids among 16,324 individuals. *Nat. Commun.* **9**, 1–12 (2018).
5. Van der Auwera, G. A. *et al.* From FastQ data to high confidence variant calls: the Genome Analysis Toolkit best practices pipeline. *Curr. Protoc. Bioinforma.* **43**, 11.10.1-33 (2013).
6. Esslinger, U. *et al.* Exome-wide association study reveals novel susceptibility genes to sporadic dilated cardiomyopathy. *PLOS ONE* **12**, e0172995 (2017).
7. Kanai, M. *et al.* Genetic analysis of quantitative traits in the Japanese population links cell types to complex human diseases. *Nat. Genet.* **50**, 390 (2018).
8. Meder, B. *et al.* A genome-wide association study identifies 6p21 as novel risk locus for dilated cardiomyopathy. *Eur. Heart J.* **35**, 1069–1077 (2014).
9. Villard, E. *et al.* A genome-wide association study identifies two loci associated with heart

failure due to dilated cardiomyopathy. *Eur. Heart J.* **32**, 1065–1076 (2011).

10. Wild, P. S. *et al.* Large-scale genome-wide analysis identifies genetic variants associated with cardiac structure and function. *J. Clin. Invest.* **127**, 1798–1812 (2017).
11. Aung Nay *et al.* Genome-Wide Analysis of Left Ventricular Image-Derived Phenotypes Identifies Fourteen Loci Associated With Cardiac Morphogenesis and Heart Failure Development. *Circulation* **140**, 1318–1330 (2019).
