# Supplementary material for: Analysis of cardiac magnetic resonance imaging in 36,000 individuals yields genetic insights into dilated cardiomyopathy
Source: Nat Commun. 2020 May 7;11:2254. doi: 10.1038/s41467-020-15823-7 (PMC7206184; doi:10.1038/s41467-020-15823-7)
Supplement: Supplementary file 3 — Reporting Summary [file 41467_2020_15823_MOESM3_ESM.pdf]

nature research

Corresponding author(s): James P Pirruccello, MD; Krishna G Aragam, MD, MS

Last updated by author(s): Mar 11, 2020

Reporting Summary

Nature Research wishes to improve the reproducibility of the work that we publish. This form provides structure for consistency and transparency in reporting. For further information on Nature Research policies, see Authors & Referees and the Editorial Policy Checklist .

Statistics

For all statistical analyses, confirm that the following items are present in the figure legend, table legend, main text, or Methods section.

n/a	Confirmed	
☐	☒	The exact sample size (n) for each experimental group/condition, given as a discrete number and unit of measurement
☐	☒	A statement on whether measurements were taken from distinct samples or whether the same sample was measured repeatedly
☐	☒	The statistical test(s) used AND whether they are one- or two-sided<br>*Only common tests should be described solely by name; describe more complex techniques in the Methods section.*
☐	☒	A description of all covariates tested
☐	☒	A description of any assumptions or corrections, such as tests of normality and adjustment for multiple comparisons
☐	☒	A full description of the statistical parameters including central tendency (e.g. means) or other basic estimates (e.g. regression coefficient) AND variation (e.g. standard deviation) or associated estimates of uncertainty (e.g. confidence intervals)
☐	☒	For null hypothesis testing, the test statistic (e.g. F, t, r) with confidence intervals, effect sizes, degrees of freedom and P value noted<br>*Give P values as exact values whenever suitable.*
☒	☐	For Bayesian analysis, information on the choice of priors and Markov chain Monte Carlo settings
☒	☐	For hierarchical and complex designs, identification of the appropriate level for tests and full reporting of outcomes
☐	☒	Estimates of effect sizes (e.g. Cohen's d, Pearson's r), indicating how they were calculated

Our web collection on statistics for biologists contains articles on many of the points above.

Software and code

Policy information about availability of computer code

Data collection	All data were provided by the UK Biobank.
Data analysis	The GWAS was performed with BOLT-LMM v2.3.2. GWAS postprocessing was performed with FUMA v1.3.4b. For permutation testing of Mendelian cardiomyopathy genes, SNPs were chosen with SNPSnap with latest major update "March 10th, 2015." Statistical analyses were otherwise conducted with R version 3.4.4.

For manuscripts utilizing custom algorithms or software that are central to the research but not yet described in published literature, software must be made available to editors/reviewers. We strongly encourage code deposition in a community repository (e.g. GitHub). See the Nature Research guidelines for submitting code & software for further information.

Data

Policy information about availability of data

All manuscripts must include a data availability statement. This statement should provide the following information, where applicable:

- Accession codes, unique identifiers, or web links for publicly available datasets
- A list of figures that have associated raw data
- A description of any restrictions on data availability

The raw UK Biobank data is made available to researchers from universities and other research institutions with genuine research inquiries, following IRB and UK Biobank approval. Individual-level sequence data for TOPMed: MESA whole genomes are available through restricted access via the TOPMed dbGaP Exchange Area (accession number phs001416.v1.p1 [https://www.ncbi.nlm.nih.gov/projects/gap/cgi-bin/study.cgi?study_id=phs001416.v1.p1]). The full GWAS summary statistics from the main analysis for each of the seven traits are available for download from the Broad Institute Cardiovascular Disease Knowledge Portal under the 'Downloads' tab at http://www.broadcvdi.org/ . The PheCode PheWAS results and the TWAS results are available in Supplementary Data Table 5. Precomputed GTEx v7 expression reference weights used for TWAS are available at http://gusevlab.org/projects/fusion/ .

Field-specific reporting

Please select the one below that is the best fit for your research. If you are not sure, read the appropriate sections before making your selection.

- [x] Life sciences
- [ ] Behavioural & social sciences
- [ ] Ecological, evolutionary & environmental sciences

For a reference copy of the document with all sections, see nature.com/documents/nr-reporting-summary-flat.pdf

Life sciences study design

All studies must disclose on these points even when the disclosure is negative.

Sample size	The Flow Diagram in Supplementary Figure 1 reveals the process of starting with the full set of participants in the UK Biobank with cardiac MRI (31,931), removing samples with anatomical mistracing, failing genotyping QC, having cardiovascular disease at baseline, or being extreme outliers, yielding 36,041 samples in the final analysis.
Data exclusions	Because the cardiac MRI volume and function parameters were derived from automated tracings, which are known to produce erroneous tracings, we manually excluded samples with tracings that did not follow the cardiac contours correctly. This aspect of the analysis plan was retrospective, initiated after observing that some samples were reported to have left ventricular volumes larger than that of an entire human thorax. We inspected and excluded mistraced samples outside of 1.5 IQR below the first or above the third quartile for each cardiac trait.
Replication	Replication was performed within MESA, a separate cohort with cardiac MRI data and whole-genome sequencing.
Randomization	Samples were not randomized. Analyses were adjusted for age at the time of enrollment into the UK Biobank or at the time of cardiac MRI, as well as sex, principal components of ancestry, the MRI scanner's identifier, and the genotyping array.
Blinding	During the process of identifying mistraced cardiac MRIs, the investigators were blinded as to disease status and cardiac MRI measurements.

Reporting for specific materials, systems and methods

We require information from authors about some types of materials, experimental systems and methods used in many studies. Here, indicate whether each material, system or method listed is relevant to your study. If you are not sure if a list item applies to your research, read the appropriate section before selecting a response.

Materials & experimental systems

n/a	Involved in the study	
x		Antibodies
x		Eukaryotic cell lines
x		Palaeontology
x		Animals and other organisms
	x	Human research participants
x		Clinical data

Methods

n/a	Involved in the study	
x		ChIP-seq
x		Flow cytometry
x		MRI-based neuroimaging

Human research participants

Policy information about studies involving human research participants

Population characteristics	As described in Supplementary Data File 2, the 36,041 participants were 52.9% female, 98% European ancestry, 64 years of age on average. 33% had used tobacco in the past. Participants with coronary artery disease, dilated cardiomyopathy, or heart failure were excluded from the study.
Recruitment	Individuals aged 40-69 in the UK were recruited via mailer from 2006-2010. As with many cohorts, there is survival bias (must have survived to age 40 to be recruited), as well as a bias towards women and healthy individuals. Participants chosen to undergo cardiac MRI are reported by the UK Biobank to have been chosen at random.
Ethics oversight	Partners Health Care institutional review board

Note that full information on the approval of the study protocol must also be provided in the manuscript.
