# Supplementary material for: Analysis of cardiac magnetic resonance imaging in 36,000 individuals yields genetic insights into dilated cardiomyopathy
Source: Nat Commun. 2020 May 7;11:2254. doi: 10.1038/s41467-020-15823-7 (PMC7206184; doi:10.1038/s41467-020-15823-7)
Supplement: Supplementary file 4 — Description of Additional Supplementary Files [file 41467_2020_15823_MOESM4_ESM.pdf]

Title: Supplementary Data File 1
Description: Genome-wide significant loci from genetic association analyses of cardiac MRI phenotypes. Loc ID: Each distinct genomic locus is labeled with a number from 1-40 for tracking across phenotypes. dbSNP: For variants which were assigned an rsID by dbSNP as of version 151, that rsID is listed. Ch: Chromosome. EAF: Effect allele frequency. INFO: Information score provided by the UK Biobank describing the quality of imputation at each locus from IMPUTE2. This is set to a value of 1 for directly genotyped SNPs. P value: The BOLT-LMM P value. Nearest Gene: Gene closest to the lead SNP. TWAS Gene: Gene within 500kb of the lead SNP with the lowest TWAS P value at the locus (if any). Cardiac Disease Genes: Genes within 500kb of the lead SNP that have previously been linked to DCM or another Mendelian cardiomyopathy from the combined gene panel in Supplementary Table 5. Prior: Symbols represent prior studies that had linked the locus to an analogous echocardiographic trait (K = Kanai, et al; W = Wild, et al; A = Aung, et al) or dilated cardiomyopathy (V = Villard, et al; E = Esslinger, et al; M = Meder, et al)(Aung Nay et al., 2019; Esslinger et al., 2017, 2017; Kanai et al., 2018; Meder et al., 2014; Wild et al., 2017). In total, 57 loci are identified, of which 12 are annotated as having previously been associated with cardiac structure and function or dilated cardiomyopathy. The effect size and standard error are dimensionless due to the inverse normal transform; a value of 1 represents 1 standard deviation from the mean.

Title: Supplementary Data File 2
Description: 96 curated disease phenotype definitions.

Title: Supplementary Data File 3
Description: Clinical characteristics of UK Biobank participants with cardiac MRI data.

Title: Supplementary Data File 4
Description: External validation. Trait: Trait tested for replication. CHR: Chromosome. BP HG19: position in Hg19 coordinates. EAF: Effect allele frequency in the UK Biobank primary GWAS. MESA: MultiEthnic Study of Atherosclerosis. In the “Sign” columns, a “+” (with cell shaded blue) represents that the effect direction in the UK Biobank primary GWAS for the trait being tested at the SNP was in the same direction as that of the effect in the other cohort; “-” (with cell shaded red) represents that the effects were in opposite directions. Cells without entries (unshaded) represent cells where a matching SNP could not be identified within the external cohort for the trait specified.

Title: Supplementary Data File 5
Description: TWAS and PheWAS results.

Title: Supplementary Data File 6
Description: Comparison of all genome-wide significant loci from the main analysis in the European-specific analysis. This table contains the same loci as in Supplementary Data File 1. In addition, the lead SNP effect estimates and P values from the European-specific analysis are provided for direct comparison. BP is the base pair distance, keyed to GRCh37. A1 is the effect allele, A0 is the non-effect allele. A1 Freq Euro represents the effect allele frequency in the Europeanspecific analysis. BETA Euro and P Euro, respectively, represent the effect size and P value in the European-specific analysis.
