# Supplementary material for: Analysis of cardiac magnetic resonance imaging in 36,000 individuals yields genetic insights into dilated cardiomyopathy
Source: Nat Commun. 2020 May 7;11:2254. doi: 10.1038/s41467-020-15823-7 (PMC7206184; doi:10.1038/s41467-020-15823-7)
Supplement: Supplementary file 5 — Supplementary Dataset 1 [file 41467_2020_15823_MOESM5_ESM.docx]

## Supplementary Data File 1: 96 curated disease phenotype definitions

| **Disease phenotype** | **Definition** |
| --- | --- |
| Hypertrophic cardiomyopathy | Self-reported history of hypertrophic cardiomyopathy during a verbal interview with a trained nurse; **or** hospitalization or death due to an ICD-10 code for hypertrophic cardiomyopathy (I42.1, I42.2); **or** hospitalization due to an ICD-9 code for hypertrophic cardiomyopathy (425.11, 425.18). |
| Heart failure | Self-reported history of heart failure or cardiomyopathy during verbal interview with trained nurse; **or** hospitalization for or death due to ICD-10 code for hypertensive heart disease, cardiomyopathy or heart failure (I11.0, I13.0, I13.2, I25.5, I42.0, I42.5, I42.8, I42.9, I50.0, I50.1, I50.9); **or** hospitalization due to ICD-9 code for heart failure or other primary cardiomyopathies (4254, 4280, 4281, 4289); **excluding** individuals with history of hypertrophic cardiomyopathy during verbal interview with trained nurse, or hospitalization for or death due to ICD-10 code for hypertrophic cardiomyopathy (I42.1, I42.2) |
| Nonischemic dilated cardiomyopathy | Hospitalization for or death due to ICD-10 code for dilated cardiomyopathy (I42.0); **excluding** individuals with history of coronary artery disease (as defined below), or history of hypertrophic cardiomyopathy during verbal interview with trained nurse, or hospitalization for or death due to ICD-10 code for hypertrophic cardiomyopathy (I42.1, I42.2) |
| Coronary artery disease | Self-reported history of myocardial infarction (MI), coronary artery bypass grafting, coronary artery angioplasty or triple heart bypass during verbal interview with trained nurse; **or** hospitalization for or death due to ICD-10 code for acute or subsequent myocardial infarction (I21, I22, I23, I24.1, I25.2); **or** hospitalization due to ICD-9 code for myocardial infarction (410, 411, 412); **or** hospitalization due to OPCS-4 code for coronary artery bypass grafting (K40, K41, K44, K45, K46), coronary endarterectomy (K47.1), or coronary angioplasty ± stenting (K49, K50.2, K75) |
| Stroke | Self-reported history of stroke during verbal interview with trained nurse; **or** hospitalization with or death due to ICD-10 code for nontraumatic subarachnoid hemorrhage, nontraumatic intracerebral hemorrhage, cerebral infarction, or unspecified stroke (I60-64); **or** hospitalization with or death due to ICD-9 code for subarachnoid hemorrhage, intracerebral hemorrhage, occlusion of cerebral arteries, or acute cerebrovascular disease (430, 431, 434, 436), as adjudicated centrally by the UK Biobank (<http://biobank.ctsu.ox.ac.uk/crystal/refer.cgi?id=462>) |
| Ischemic stroke | Self-reported history of ischemic stroke during verbal interview with trained nurse; **or** hospitalization with or death due to ICD-10 code for cerebral infarction, or unspecified stroke (I63, 64); **or** hospitalization with or death due to ICD-9 code for occlusion of cerebral arteries or acute cerebrovascular disease (434, 436), as adjudicated centrally by the UK Biobank (<http://biobank.ctsu.ox.ac.uk/crystal/refer.cgi?id=462>) |
| Intracerebral hemorrhage | Self-reported history of brain hemorrhage during verbal interview with trained nurse; **or** hospitalization with or death due to ICD-10 code for nontraumatic intracerebral hemorrhage (I61); **or** hospitalization with or death due to ICD-9 code for intracerebral hemorrhage (431), as adjudicated centrally by the UK Biobank (<http://biobank.ctsu.ox.ac.uk/crystal/refer.cgi?id=462>) |
| Subarachnoid hemorrhage | Self-reported history of subarachnoid hemorrhage during verbal interview with trained nurse; **or** hospitalization with or death due to ICD-10 code for nontraumatic subarachnoid hemorrhage (I60); **or** hospitalization with or death due to ICD-9 code for subarachnoid hemorrhage (430), as adjudicated centrally by the UK Biobank (<http://biobank.ctsu.ox.ac.uk/crystal/refer.cgi?id=462>) |
| Pulmonary hypertension | Hospitalization with or death due to ICD-10 code for primary or other secondary pulmonary hypertension (I27.0, I27.2); **or** hospitalization with ICD-9 code for primary pulmonary hypertension (4160) |
| Atrial fibrillation or flutter | Self-reported history of atrial fibrillation, atrial flutter, or cardioversion during verbal interview with trained nurse; **or** hospitalization with or death due to ICD-10 code for atrial fibrillation or atrial flutter (I48); **or** hospitalization with ICD-9 code for atrial fibrillation or atrial flutter (4273); **or** hospitalization with OPCS-4 code for percutaneous transluminal ablation (K57.1, K 62.1, K62.2, K62.3, K62.4) |
| Venous thromboembolism | Self-reported history of venous thromboembolic disease, pulmonary embolism or deep venous thrombosis during verbal interview with trained nurse; **or** hospitalization with or death due to ICD-10 code for pulmonary embolism (I26), phlebitis or thrombophlebitis (I80.0-I80.3, I80.8-I80.9), portal vein thrombosis (I81), Budd-Chiari syndrome (I82.0), or other coagulation defects (D68); **or** hospitalization with ICD-9 code for pulmonary embolism or phlebitis/thrombophlebitis (4151, 4511); **or** hospitalization with OPCS-4 code for insertion of inferior vena cava filter or open thrombectomy of lower extremity vein (L79.1, L90.2) |
| Peripheral artery disease | Self-reported history of peripheral vascular disease, arterial embolism, intermittent claudication, leg artery bypass, leg artery angioplasty, or leg amputation during verbal interview with trained nurse; **or** hospitalization with or death due to ICD-10 code for atherosclerosis of (non-coronary) arteries or peripheral vascular disease (I70.0, I70.00, I70.01, I70.2, I70.20, I70.21, I70.8, I70.80, I70.9, I70.90, I73.8 or I73.9); **or** hospitalization with ICD-9 code for atherosclerosis of arteries or peripheral vascular disease (4400, 4402, 4438, 4439); **or** hospitalization with OPCS-4 coded procedure for leg amputation, or leg artery procedure such as bypass, stent or angioplasty (X09.3-09.5, L21.6, L51.3, L51.6, L51.8, L52.1, L52.2, L54.1, L54.4, L54.8, L59.1-L59.8, L60.1, L60.2, L63.1, L63.5, L63.9, L66.7) |
| Hypertension | Self-reported history of hypertension, essential hypertension or high blood pressure during verbal interview with trained nurse; **or** hospitalization with or death due to ICD-10 code for essential hypertension, hypertensive heart disease, hypertensive renal disease, or secondary hypertension (I10, I11, I12, I13, I15); **or** hospitalization with ICD-9 code for essential hypertension, hypertensive heart disease, hypertensive renal disease, or secondary hypertension (401, 402, 403, 404, 405) |
| Hypercholesterolemia | Self-reported history of high cholesterol during verbal interview with trained nurse; **or** hospitalization with or death due to ICD-10 code for hypercholesterolemia, hypertriglyceridemia, or hyperlipidemia (E78.0-E78.2, E78.4, E78.5) |
| Supraventricular arrhythmia –general inclusive definition | Self-reported history of Wolff-Parkinson-White syndrome or supraventricular tachycardia during verbal interview with trained nurse; **or** hospitalization with or death due to ICD-10 code for Preexcitation syndrome, supraventricular tachycardia, atrial premature depolarization (I45.6, I47.1, I49.1); **or** hospitalization with ICD-9 code for anomalous atrioventricular excitation or paroxysmal supraventricular tachycardia (4267, 4270); **or** hospitalization with OPCS-4 coded procedure for open division of accessory pathway, percutaneous transluminal ablation of accessory pathway/atrial wall/conduction system (K52.4, K57.2, K57.4, K57.5) |
| Supraventricular arrhythmia –Wolff-Parkinson-White syndrome | Self-reported history of Wolff-Parkinson-White syndrome during verbal interview with trained nurse; **or** hospitalization with or death due to ICD-10 code for Preexcitation syndrome (I45.6); **or** hospitalization with ICD-9 for to anomalous atrioventricular excitation (4267); **or** hospitalization with OPCS-4 coded procedure for open division of accessory pathway, percutaneous transluminal ablation of accessory pathway (K52.4, K57.4) |
| Supraventricular arrhythmia –premature atrial contractions | Hospitalization with or death due to ICD-10 code for atrial premature depolarization (I49.1) |
| Supraventricular arrhythmia –supraventricular tachycardia | Self-reported history of supraventricular tachycardia during verbal interview with trained nurse; **or** hospitalization with or death due to ICD-10 code for supraventricular tachycardia (I47.1); **or** hospitalization with ICD-9 code for paroxysmal supraventricular tachycardia (4270); **or** hospitalization with OPCS-4 coded procedure for percutaneous transluminal ablation of atrial wall/conduction system (K57.2, K57.5) |
| Bradyarrhythmia – general inclusive definition | Self-reported history of sick sinus syndrome, pacemaker/defibrillator insertion, or pacemaker battery change during verbal interview with trained nurse; **or** hospitalization with or death due to ICD-10 code for atrioventricular and intraventricular block or sick sinus syndrome (I44, I45.0-I45.5, I49.5); **or** hospitalization with ICD-9 code for atrioventricular or intraventricular block (4260, 4261, 4263, 4264, 4265, 4266); **or** hospitalization with OPCS-4 coded procedure for cardiac pacemaker system (K60, K61) |
| Bradyarrhythmia – sinus node dysfunction | Self-reported history of sick sinus syndrome during verbal interview with trained nurse; **or** hospitalization with or death due to ICD-10 code for sick sinus syndrome (I49.5) |
| Bradyarrhythmia - AV block / distal conduction disease | Hospitalization with or death due to ICD-10 code for atrioventricular and intraventricular block (I44, I45.0-I45.5); **or** hospitalization with ICD-9 code for atrioventricular or intraventricular block (4260, 4261, 4263, 4264, 4265, 4266) |
| Bradyarrhythmia - Pacemaker | Self-reported history of pacemaker/defibrillator insertion, or pacemaker battery change during verbal interview with trained nurse; **or** hospitalization with OPCS-4 coded procedure for cardiac pacemaker system (K60, K61) |
| Implantable cardioverter defibrillator | Hospitalization with OPCS-4 coded procedure for cardioverter defibrillator introduced through the vein (K59) |
| Ventricular arrhythmia - General inclusive definition | Hospitalization with or death due to ICD-10 code for ventricular arrhythmias, ventricular premature depolarization or cardiac arrest (I46.0, I46.1, I46.9, I47.0, I47.2, I49.0, I49.3); **or** hospitalization with ICD-9 code for ventricular arrhythmias or cardiac arrest (4271, 4274, 4275); **or** hospitalization with OPCS-4 coded procedure for percutaneous transluminal/radiofrequency ablation (K57.6, K64.1) or resuscitation/defibrillation (X50.3, X50.4, X50.8, X50.9) |
| Ventricular arrhythmia – Ventricular tachycardia | Hospitalization with or death due to ICD-10 code for ventricular arrhythmias (I47.0, I47.2, I49.0); **or** hospitalization with ICD-9 code for ventricular arrhythmias (4271, 4274); **or** hospitalization with OPCS-4 coded procedure for percutaneous transluminal/radiofrequency ablation (K57.6, K64.1) |
| Ventricular arrhythmia – Ventricular premature depolarizations | Hospitalization with or death due to ICD-10 code for ventricular premature depolarization (I49.3) |
| Ventricular arrhythmia – Sudden cardiac death | Hospitalization with or death due to ICD-10 code for cardiac arrest (I46.0, I46.1, I46.9); **or** hospitalization with ICD-9 code for ventricular arrhythmias or cardiac arrest (4275); **or** hospitalization with OPCS-4 coded procedure for resuscitation/defibrillation (X50.3, X50.4, X50.8, X50.9) |
| Aortic valve disease | Self-reported history of aortic stenosis, aortic valve disease, aortic regurgitation or aortic valve repair/replacement during verbal interview with trained nurse; **or** hospitalization with or death due to ICD-10 code for rheumatic aortic valve disease (I06), unspecified aortic valve disorders (I08.0, I08.2, I08.3, I39.1), or nonrheumatic aortic valve disorders (I35); **or** hospitalization with ICD-9 code for rheumatic aortic insufficiency (3951), or unspecified diseases of aortic valve (3959, 4241); **or** hospitalization with OPCS-4 code for aortic valve repair/revision (K26, K30.2) |
| Aortic stenosis | Self-reported history of aortic stenosis during verbal interview with trained nurse; **or** hospitalization with or death due to ICD-10 code for rheumatic aortic stenosis (I06.0, I06.2) or nonrheumatic aortic stenosis (I35.0, I35.2) |
| Aortic regurgitation | Self-reported history of aortic regurgitation during verbal interview with trained nurse; **or** hospitalization with or death due to ICD-10 code for rheumatic aortic insufficiency (I06.1, I06.2) or nonrheumatic aortic insufficiency (I35.0, I35.2); **or** hospitalization with ICD-9 code for rheumatic aortic insufficiency (3951) |
| Mitral valve disease | Self-reported history of mitral valve disease, mitral stenosis, mitral valve prolapse, mitral regurgitation, or mitral valve repair/replacement during verbal interview with trained nurse; **or** hospitalization with or death due to ICD-10 code for rheumatic mitral valve diseases (I05), nonrheumatic mitral valve diseases (I34), or unspecified mitral valve disorders (I08.0, I08.1, I08.3, I39.0); **or** hospitalization with ICD-9 code for mitral stenosis + insufficiency (3940, 3942), unspecified diseases of mitral valve (3949, 4240); **or** hospitalization with OPCS-4 code for mitral valve repair/revision (K25, K30.1), mitral valve annuloplasty (K34.1), percutaneous transluminal mitral valvotomy (K35.1) |
| Mitral stenosis | Self-reported history of mitral stenosis during verbal interview with trained nurse; **or** hospitalization with or death due to ICD-10 code for rheumatic and nonrheumatic mitral stenosis (I05.0, I34.2); **or** hospitalization with ICD-9 code for mitral stenosis + insufficiency (3940, 3942); **or** hospitalization with OPCS-4 code for percutaneous transluminal mitral valvotomy (K35.1) |
| Mitral regurgitation | Self-reported history of mitral regurgitation during verbal interview with trained nurse; **or** hospitalization with or death due to ICD-10 code for rheumatic and nonrheumatic mitral insufficiency (I05.1, I05.2, I34.0); **or** hospitalization with ICD-9 code for mitral insufficiency (3942); **or** hospitalization with OPCS-4 code for mitral valve annuloplasty (K34.1) |
| Mitral valve prolapse | Self-reported history of mitral valve prolapse during verbal interview with trained nurse; **or** hospitalization with or death due to ICD-10 code for mitral valve prolapse (I34.1) |
| Tricuspid valve disease | Hospitalization with or death due to ICD-10 code for rheumatic tricuspid valve diseases (I07), nonrheumatic tricuspid valve diseases (I36), or unspecified tricuspid valve disorders (I08.1, I08.2, I08.3); **or** hospitalization with OPCS-4 code for tricuspid valve repair/revision (K27, K30.3), or tricuspid valve annuloplasty (K34.2) |
| Tricuspid stenosis | Hospitalization with or death due to ICD-10 code for rheumatic and nonrheumatic tricuspid stenosis (I07.0, I07.2, I36.0) |
| Tricuspid regurgitation | Hospitalization with or death due to ICD-10 code for rheumatic and nonrheumatic tricuspid regurgitation (I07.1, I07.2, I36.1); **or** hospitalization with OPCS-4 code for tricuspid valve annuloplasty (K34.2) |
| Pulmonary valve disease | Hospitalization with or death due to ICD-10 code for pulmonary valve disorders (I37, I39.3); **or** hospitalization with ICD-9 code for pulmonary valve disorders (4243); **or** hospitalization with OPCS-4 code for pulmonary valve repair/revision (K28, K30.4), or percutaneous transluminal pulmonary valve replacement (K35.7) |
| Pulmonary stenosis | Hospitalization with or death due to ICD-10 code for pulmonary stenosis (I37.0, I37.2) |
| Pulmonary regurgitation | Hospitalization with or death due to ICD-10 code for pulmonary insufficiency (I37.1, I37.2) |
| Valvular disease, unspecified | Self-reported history of heart valve problem/murmur/surgery or other valve repair/replacement during verbal interview with trained nurse; **or** hospitalization with or death due to ICD-10 code for multiple and/or unspecified rheumatic and nonrheumatic valve diseases (I08.8, I08.9, I09.1, I39.4); **or** hospitalization with OPCS-4 code for unspecified valve replacement/repair/procedure (K29, K34.3, K35.8) |
| Congenital heart disease | Hospitalization with or death due to ICD-10 code for congenital malformations of cardiac chambers/connexions, cardiac septa, aortic/mitral/tricuspid/pulmonary valves, and other/unspecified malformation (Q20.1, Q20.2, Q20.3, Q20.4, Q20.5, Q20.6, Q20.8, Q20.9; Q21, Q22, Q23, Q24); **or** hospitalization with ICD-9 code for congenital anomalies of the heart and cardiac septa (745, 746); **or** hospitalization with OPCS-4 code for repair of tetralogy of Fallot (K04), correction of total anomalous pulmonary venous connection (K07), repair of defect of atrioventricular/interatrial/interventricular/unspecified septum (K09, K10, K11, K12), transluminal repair of septal defect (K13), transluminal repair of atrial septum or patent oval foramen with prosthesis (K16.3, K16.5), creation of valved/other cardiac conduit (K18, K19, refashioning of atrium (K20) |
| Congenital heart disease – Ebstein’s anomaly | Hospitalization with or death due to ICD-10 code for Ebstein’s anomaly (Q22.5) |
| Diabetes mellitus, type 1 | Self-reported history of Type 1 diabetes during verbal interview with trained nurse; **or** hospitalization with or death due to ICD-10 code for insulin-dependent diabetes mellitus (E10) |
| Diabetes mellitus, type 2 | Self-reported history of type 2 diabetes during verbal interview with trained nurse; **or** hospitalization with or death due to ICD-10 code for non-insulin-dependent diabetes mellitus (E11) |
| Diabetes mellitus, all | Self-reported history of diabetes, gestational diabetes, type 1/type 2 or insulin use during verbal interview with trained nurse; **or** hospitalization with or death due to ICD-10 code for insulin-dependent and non-insulin-dependent diabetes mellitus (E10, E11), malnutrition-related diabetes mellitus (E12), other specified/unspecified diabetes mellitus (E13, E14); **or** hospitalization with ICD-9 code for diabetes mellitus with/without complications (2500, 2501, 2503, 2504, 2505, 2509) |
| Hyperthyroidism | Self-reported history of hyperthyroidism/thyrotoxicosis during verbal interview with trained nurse; **or** hospitalization with or death due to ICD-10 code for hyperthyroidism/thyrotoxicosis (E05); **or** hospitalization with ICD-9 code for thyrotoxicosis with/without goiter (242) |
| Hypothyroidism | Self-reported history of hypothyroidism/myxoedema during verbal interview with trained nurse; **or** hospitalization with or death due to ICD-10 code for hypothyroidism (E03); **or** hospitalization with ICD-9 code for acquired hypothyroidism (244) |
| Gout | Self-reported history of gout during verbal interview with trained nurse; **or** hospitalization with or death due to ICD-10 code for gout (M10.0, M10.2, M10.3, M10.4, M10.9); **or** hospitalization with ICD-9 code for gout (274) |
| Enlarged prostate | Self-reported history of enlarged prostate during verbal interview with trained nurse; **or** hospitalization with or death due to ICD-10 code for hyperplasia of prostate (N40); **or** hospitalization with ICD-9 code for hyperplasia of prostate (600) |
| Uterine fibroids | Self-reported history of uterine fibroids or myomectomy/fibroid removal during verbal interview with trained nurse; **or** hospitalization with or death due to ICD-10 code for leiomyoma of uterus (D25); **or** hospitalization with ICD-9 code for uterine leiomyoma (218) |
| Chronic kidney disease | Self-reported history of kidney failure ± dialysis, kidney nephropathy, IgA nephropathy, diabetic nephropathy or kidney transplant during verbal interview with trained nurse; **or** hospitalization with or death due to ICD-10 code for hypertensive renal disease, chronic renal failure, end stage renal failure or chronic kidney disease (I12.0, I13.1, I13.2, N18, N18.0-18.5, N18.8, N18.9); **or**  hospitalization with ICD-9 code due to chronic renal failure (585, 5859); **or**  hospitalization with OPCS-4 coded procedure for kidney transplantation (M01.1-01.5, M01.8, M01.9) |
| Gastroesophageal reflux disease | Self-reported history of gastroesophageal/gastric reflux during verbal interview with trained nurse; **or** hospitalization with or death due to ICD-10 code for gastroesophageal reflux disease (K21); **or** hospitalization with ICD-9 code for esophageal reflux (53010, 53011) |
| Irritable bowel syndrome | Self-reported history of irritable bowel syndrome during verbal interview with trained nurse; **or** hospitalization with or death due to ICD-10 code for irritable bowel syndrome (K58); **or** hospitalization with ICD-9 code for irritable colon (5641) |
| Cholelithiasis | Self-reported history of cholelithiasis/gallstones or gallstone removal during verbal interview with trained nurse; **or** hospitalization with or death due to ICD-10 code for gallstone ileus (K56.3) or cholelithiasis (K80; **or** hospitalization with ICD-9 code for cholelithiasis (574); **or** hospitalization with OPCS-4 coded procedure for open removal/percutaneous dissolution/fragmentation of gall bladder calculus (J21.1, J24.2, J24.3, J26.1) |
| Inflammatory bowel disease | Self-reported history of inflammatory bowel disease, Crohn’s disease, or ulcerative colitis during verbal interview with trained nurse; **or** hospitalization with or death due to ICD-10 code for Crohn’s disease or ulcerative colitis (K50, K51); **or** hospitalization with ICD-9 code for regional enteritis, idiopathic proctocolitis (555, 556) |
| Crohn’s disease | Self-reported history of Crohn’s disease during verbal interview with trained nurse; **or** hospitalization with or death due to ICD-10 code for Crohn’s disease (K50); **or** hospitalization with ICD-9 code for regional enteritis (555) |
| Ulcerative colitis | Self-reported history of ulcerative colitis during verbal interview with trained nurse; **or** hospitalization with or death due to ICD-10 code for ulcerative colitis (K51); **or** hospitalization with ICD-9 code for idiopathic proctocolitis (556) |
| Diverticular disease | Self-reported history of diverticular disease during verbal interview with trained nurse; **or** hospitalization with or death due to ICD-10 code for diverticular disease of intestine (K57); **or** hospitalization with ICD-9 code for diverticula of intestine (562) |
| Pancreatitis | Self-reported history of pancreatitis during verbal interview with trained nurse; **or** hospitalization with or death due to ICD-10 code for acute pancreatitis (K85) or alcohol-induced/other chronic pancreatitis (K86.0, K86.1); **or** hospitalization with ICD-9 code for acute/chronic pancreatitis (5770, 5771) |
| Migraine | Self-reported history of migraine during verbal interview with trained nurse; **or** hospitalization with or death due to ICD-10 code for migraine (G43) |
| Depression | Self-reported history of depression during verbal interview with trained nurse; **or** hospitalization with or death due to ICD-10 code for depressive episode or recurrent depressive disorder (F32, F33); **or** hospitalization with ICD-9 code for depressive disorder (3119) |
| Bipolar disorder | Self-reported history of mania/bipolar disorder/manic depression during verbal interview with trained nurse; **or** hospitalization with or death due to ICD-10 code for bipolar affective disorder (F31) |
| Anxiety | Self-reported history of anxiety/panic attacks during verbal interview with trained nurse; **or** hospitalization with or death due to ICD-10 code for anxiety disorders (F41) |
| Schizophrenia | Self-reported history of schizophrenia during verbal interview with trained nurse; **or** hospitalization with or death due to ICD-10 code for schizophrenia (F20) |
| Post-traumatic stress disorder | Self-reported history of post-traumatic stress disorder during verbal interview with trained nurse; **or** hospitalization with or death due to ICD-10 code for post-traumatic stress disorder (F43.1) |
| Multiple sclerosis | Self-reported history of multiple sclerosis during verbal interview with trained nurse; **or** hospitalization with or death due to ICD-10 code for multiple sclerosis (G35); **or** hospitalization with ICD-9 code for multiple sclerosis (3409) |
| Parkinson’s disease | Self-reported history of Parkinson’s disease during verbal interview with trained nurse; **or** hospitalization with or death due to ICD-10 code for Parkinson’s disease (G20) or dementia in Parkinson’s disease (F02.3); **or** hospitalization with ICD-9 code for paralysis agitans (3320) |
| Epilepsy | Self-reported history of epilepsy during verbal interview with trained nurse; **or** hospitalization with or death due to ICD-10 code for epilepsy (G40); **or** hospitalization with ICD-9 code for epilepsy (3450, 3451, 3452, 3454, 3459) |
| Alzheimer’s / Dementia | Self-reported history of dementia/Alzheimer’s/cognitive impairment during verbal interview with trained nurse; **or** hospitalization with or death due to ICD-10 code for dementia in Alzheimer’s disease (F00) |
| Back pain | Self-reported history of back pain or sciatica during verbal interview with trained nurse; **or** hospitalization with or death due to ICD-10 code for Dorsalgia (M54); **or** hospitalization with ICD-9 code for cervicalgia, spinal stenosis, pain in thoracic spine, lumbago, sciatica, thoracic or lumbosacral neuritis/radiculitis, unspecified backache (7231, 7240, 7241, 7242, 7243, 7244, 7245) |
| Sciatica | Self-reported history of sciatica during verbal interview with trained nurse; **or** hospitalization with or death due to ICD-10 code for sciatica (M54.3); **or** hospitalization with ICD-9 code for sciatica (7243) |
| Osteoporosis | Self-reported history of osteoporosis during verbal interview with trained nurse; **or** hospitalization with or death due to ICD-10 code for osteoporosis with/without pathological fracture (M80, M81); **or** hospitalization with ICD-9 code for osteoporosis (7330) |
| Intervertebral disc displacement | Self-reported history of prolapsed/slipped disc during verbal interview with trained nurse; **or** hospitalization with or death due to ICD-10 code for other cervical/intervertebral disc displacement (M50.2, M51.2); **or** hospitalization with ICD-9 code for cervical/thoracic/lumbar/unspecified intervertebral disc displacement (7220, 7221, 7222) |
| Osteoarthritis | Self-reported history of osteoarthritis during verbal interview with trained nurse; **or** hospitalization with or death due to ICD-10 code for polyarthrosis, coxarthrosis, gonarthrosis, first carpometacarpal or other arthrosis (M15, M16, M17, M18, M19); **or** hospitalization with ICD-9 code for osteoarthrosis (715) |
| Rheumatoid arthritis | Self-reported history of rheumatoid arthritis during verbal interview with trained nurse; **or** hospitalization with or death due to ICD-10 code for rheumatoid arthritis (M05, M06); **or** hospitalization with ICD-9 code for rheumatoid arthritis (714) |
| Lupus erythematosus | Self-reported history of systemic lupus erythematosus during verbal interview with trained nurse; **or** hospitalization with or death due to ICD-10 code for lupus erythematosus (L93) or systemic lupus erythematosus (M32.1, M32.8, M32.9); **or** hospitalization with ICD-9 code for lupus erythematosus (6954) |
| Sarcoidosis | Self-reported history of sarcoidosis during verbal interview with trained nurse; **or** hospitalization with or death due to ICD-10 code for sarcoidosis (D86); **or** hospitalization with ICD-9 code for sarcoidosis (135) |
| Psoriasis | Self-reported history of psoriasis during verbal interview with trained nurse; **or** hospitalization with or death due to ICD-10 code for psoriasis (L40), psoriatic arthropathy, arthritis mutilans, psoriatic spondylitis (M07.0, M07.1, M07.2, M07.3); **or** hospitalization with ICD-9 code for psoriasis/psoriatic arthropathy (6960, 6961) |
| Dermatitis | Self-reported history of eczema/dermatitis during verbal interview with trained nurse; **or** hospitalization with or death due to ICD-10 code for atopic dermatitis, seborrhoeic dermatitis, diaper dermatitis, allergic contact dermatitis, irritant/unspecified contact dermatitis (L20, L21, L22, L23, L24, L25, L26, L27, L30), Lichen simplex chronicus and prurigo (L28), pruritus (L29); **or** hospitalization with ICD-9 code for atopic/contact dermatitis (691, 692) |
| Iron deficiency anemia | Self-reported history of iron deficiency anemia during verbal interview with trained nurse; **or** hospitalization with or death due to ICD-10 code for iron deficiency anemia (D50); **or** hospitalization with ICD-9 code for iron deficiency anemia (280) |
| Asthma | Self-reported history of asthma during verbal interview with trained nurse; **or** hospitalization with or death due to ICD-10 code for asthma or status asthmaticus (J45, J46); **or** hospitalization with ICD-9 code for asthma (493) |
| Chronic obstructive pulmonary disease | Self-reported history of chronic obstructive airways/emphysema during verbal interview with trained nurse; **or** hospitalization with or death due to ICD-10 code for chronic bronchitis, emphysema, or other chronic obstructive pulmonary disease (J41, J42, J43, J44); **or** hospitalization with ICD-9 code for chronic bronchitis, emphysema or unspecified chronic airways obstruction (491, 492, 496) |
| Pneumonia | Self-reported history of pneumonia during verbal interview with trained nurse; **or** hospitalization with or death due to ICD-10 code for pneumonia (J12, J13, J14, J15, J16, J17, J18); **or** hospitalization with ICD-9 code for pneumonia (481, 482, 483, 484, 485, 486) |
| Allergic rhinitis | Self-reported history of hayfever/allergic rhinitis during verbal interview with trained nurse; **or** hospitalization with or death due to ICD-10 code for allergic rhinitis (J30.1-J30.4); **or** hospitalization with ICD-9 code for allergic rhinitis (477) |
| Sleep apnea | Self-reported history of sleep apnea during verbal interview with trained nurse; **or** hospitalization with or death due to ICD-10 code for sleep apnea (G47.3) |
| Cataract | Self-reported history of cataract during verbal interview with trained nurse; **or** hospitalization with or death due to ICD-10 code for cataract (H25, H26); **or** hospitalization with ICD-9 code for cataract (366) |
| Glaucoma | Self-reported history of glaucoma during verbal interview with trained nurse; **or** hospitalization with or death due to ICD-10 code for glaucoma (H40); **or** hospitalization with ICD-9 code for glaucoma (365) |
| Lung cancer | Self-reported history of lung cancer during verbal interview with trained nurse; **or** hospitalization with or death due to ICD-10 code for malignant neoplasm of bronchus and lung (C34); **or** hospitalization with ICD-9 code for malignant neoplasm of bronchus and lung (1629) |
| Breast cancer | Self-reported history of breast cancer during verbal interview with trained nurse; **or** hospitalization with or death due to ICD-10 code for malignant neoplasm of breast (C50); **or** hospitalization with ICD-9 code for malignant neoplasm of breast (174) |
| Colorectal cancer | Self-reported history of colorectal/sigmoid/rectal cancer during verbal interview with trained nurse; **or** hospitalization with or death due to ICD-10 code for malignant neoplasm of colon (C18.0, C18.2-18.9); **or** hospitalization with ICD-9 code for malignant neoplasm of colon/rectum (1532, 1533, 1541) |
| Skin cancer | Self-reported history of skin, malignant melanoma, non-melanoma skin cancer, or squamous cell carcinoma during verbal interview with trained nurse; **or** hospitalization with or death due to ICD-10 code for malignant melanoma or other malignant neoplasms of skin (C43, C44); **or** hospitalization with ICD-9 code for malignant melanoma/neoplasm of skin (172, 173) |
| Prostate cancer | Self-reported history of prostate cancer during verbal interview with trained nurse; **or** hospitalization with or death due to ICD-10 code for malignant neoplasm of prostate (C61) |
| Cervical cancer | Self-reported history of cervical cancer during verbal interview with trained nurse; **or** hospitalization with or death due to ICD-10 code for malignant neoplasm of cervix uteri (C53) |
| Bladder cancer | Self-reported history of bladder cancer during verbal interview with trained nurse; **or** hospitalization with or death due to ICD-10 code for malignant neoplasm of bladder (C67); **or** hospitalization with ICD-9 code for malignant neoplasm of bladder (188) |
| Gestational hypertension – preeclampsia | Self-reported history of gestational hypertension/pre-eclampsia during verbal interview with trained nurse; **or** hospitalization with or death due to ICD-10 code for pre-existing hypertensive disorder with superimposed proteinuria (O11), gestational hypertension (O13, O14), eclampsia (O15); **or** hospitalization with ICD-9 code for transient hypertension of pregnancy, preeclampsia or eclampsia (6423, 6424, 6425, 6426, 6427) |

## 
